# Targeting a Calcium‐Associated Mitochondrial Vulnerability in TET2‐Deficient Myeloid Cells

**DOI:** 10.1002/advs.78748

**Published:** 2026-11-09

**Authors:** Yue Wang, Minh Duc Pham, Despina Stefanoska, Ting Yuan, Arka Provo Das, Chaonan Zhu, Meiqian Wu, Thanh Thuy Duong, Jaskiran Kaur, Yu Hao, Johannes Krefting, Marius Schwab, Jonathan Ward, Maria Duda, Wesley Abplanalp, Moritz von Scheidt, Andreas M. Zeiher, Stefanie Dimmeler, Jaya Krishnan

**Affiliations:** ^1^ Department of Medicine III, Cardiology/Angiology/ Nephrology Goethe University Hospital Frankfurt am Main Germany; ^2^ Institute For Cardiovascular Regeneration, Centre For Molecular Medicine Goethe University Frankfurt am Main Germany; ^3^ Partner Site Frankfurt Rhine‐Main German Center for Cardiovascular Research Germany; ^4^ Genome Biologics Frankfurt am Main Germany; ^5^ College of Animal Science Jilin University Changchun China; ^6^ Department of Cardiology TUM Klinikum Deutsches Herzzentrum Technical University Munich Munich Germany; ^7^ Partner Site Munich Heart Alliance Deutsches Zentrum für Herz‐ und Kreislaufforschung (DZHK) Munich Germany; ^8^ Cardiopulmonary Institute Frankfurt Germany

**Keywords:** cardiac organoids, cardioinflammation, clonal hematopoiesis, Lanatoside C, macrophages, mitochondrial calcium signaling, TET2

## Abstract

Clonal hematopoiesis (CH) driven by loss‐of‐function mutations in TET2 is associated with increased cardiovascular risk, yet therapeutic strategies capable of preferentially targeting pathogenic *TET2*‐deficient myeloid effector cells remain poorly defined. Here, we identify a TET2 loss‐of‐function‐associated ion‐mitochondrial vulnerability that enables preferential pharmacological suppression of *TET2*‐deficient macrophages. Using chemical‐genetic profiling, we find that perturbation of ion homeostasis preferentially compromises survival of *TET2*‐deficient macrophages through increased mitochondrial‐enriched Ca^2+^‐sensitive signaling and activation of intrinsic apoptotic pathways. The cardiac glycoside Lanatoside C preferentially induces mitochondrial depolarization and apoptosis in *TET2*‐deficient macrophages, with substantially smaller effects in control cells. In multicellular human cardiac organoids, preferential reduction of *TET2*‐knockdown macrophages suppresses inflammatory remodeling, reduces cardiomyocyte apoptosis and fibrosis, and improves abnormal calcium‐handling dynamics. In a murine partial donor‐cell reconstitution model, Lanatoside C reduced relative donor‐derived monocyte and neutrophil frequencies in Tet2‐deficient donor‐cell recipients; these findings do not establish effects on stem‐cell fitness. Together, these findings identify a pharmacologically exploitable ion‐mitochondrial vulnerability associated with TET2 loss‐of‐function and establish human cardiac organoids as a platform for causal interrogation of immune‐driven cardiovascular pathology.

## Graphical abstract

Reduced TET2 function creates a calcium‐associated mitochondrial vulnerability in macrophages that is preferentially exploited by Lanatoside C. The resulting stress promotes apoptosis of TET2‐deficient macrophages, reduces inflammatory and fibrotic injury in human cardiac organoids, and highlights a genotype‐associated myeloid liability that may be therapeutically tractable in clonal hematopoiesis‐associated cardiovascular disease.

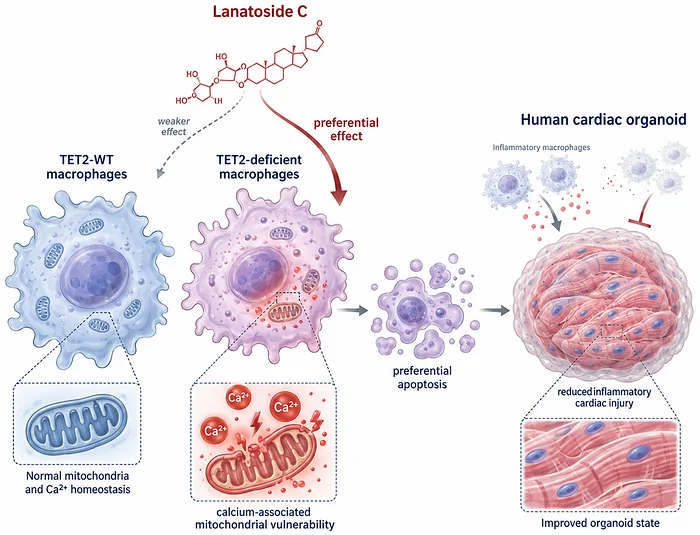

## 1 Introduction

Clonal hematopoiesis is an age‐related condition defined by the expansion of hematopoietic clones carrying somatic mutations with a variant allele frequency (VAF) of at least 2% in peripheral blood, and is present in nearly 10% of individuals older than 60 years [1, 2, 3]. These mutations, often affecting myeloid lineages, confer a proliferative advantage and promote the secretion of pro‐inflammatory cytokines [4, 5]. Epidemiological studies have linked CH to adverse cardiovascular outcomes, including myocardial infarction, coronary artery disease, and increased mortality [6, 7, 8, 9, 10]. Recent data from a large coronary artery disease cohort further demonstrated that Clonal hematopoiesis of indeterminate potential (CHIP), including TET2‐driven CHIP, is associated with increased mortality and adverse atherosclerotic phenotypes [11].

Among the most frequently mutated drivers of CH is the Tet methylcytosine dioxygenase 2 (TET2) gene, which encodes a dioxygenase required for DNA demethylation [6, 12]. Heterozygous loss‐of‐function mutations in *TET2* occur in 10% to 30% of CH cases [12, 13]. TET2 loss‐of‐function is associated with elevated interleukin‐1β (IL‐1β), interleukin‐6 (IL‐6), and tumor necrosis factor‐α (TNFα) levels in both *TET2*‐mutant individuals and murine models [2, 4, 14, 15, 16], establishing a mechanistic link between CH and cardiovascular events, including myocardial infarction, stroke, and heart failure [5, 10]. Importantly, recent large‐scale cohort analyses demonstrated that *TET2*, but not *DNMT3A* or *ASXL1*, CH was associated with higher IL‐6, adverse cardiac remodeling, and atrial fibrillation, supporting a potentially distinct cardiovascular‐inflammatory association relative to other common CH drivers [17]. Together, these observations suggest that *TET2* mutations confer not only clonal advantage but also a qualitatively distinct inflammatory and metabolic phenotype that may be uniquely targetable. However, it remains unknown whether genotype‐specific metabolic dependencies arise following TET2 loss and whether these can be pharmacologically exploited to selectively eliminate pathogenic *TET2*‐deficient myeloid effector cells.

Mechanistically, *TET2* loss alters DNA methylation landscapes, enhances self‐renewal, and biases hematopoiesis toward myeloid lineages [6, 18, 19, 20]. Consequently, these mutations foster sustained inflammatory signaling and clonal expansion, thereby amplifying cardiovascular pathology [6, 21]. Most experimental studies, however, rely on animal models or simplified in vitro systems that incompletely recapitulate the vascular and cellular complexity of human cardiac tissue, limiting mechanistic analysis of inflammatory cardiac disease.

To address the limitations of reductionist cardiac culture systems, we utilized cardiomorphs (CDMs), self‐organizing human cardiac organoids derived from pluripotent stem cells that reproduce key multicellular and functional properties of the human myocardium [22]. Through staged mesodermal patterning and cardiogenic specification, CDMs generate cardiomyocytes alongside endothelial, mural, stromal, neuronal, and immune populations that assemble into structured myocardial microtissues [22]. This multicellular architecture supports emergent vascular organization and tissue‐level functional responses not observed in conventional cardiac spheroid systems [22]. As such, CDMs enable causal interrogation of immune‐cardiac interactions in a human‐relevant context [22].

By establishing a co‐culture of control and *TET2*‐knockdown (TET2‐KD) macrophages with in vitro‐derived CDMs, we generated a multicellular human cardiac model for causal interrogation of immune‐driven cardiac dysfunction. This system captured key TET2 loss‐of‐function‐associated phenotypes, including mitochondrial dysfunction, calcium dysregulation, apoptosis, and inflammation. To systematically identify genotype‐dependent liabilities, we performed chemical‐genetic profiling of *TET2*‐deficient macrophages. This screen identified Lanatoside C as a selective tool compound that induces apoptosis in *TET2*‐KD macrophages, thereby reducing macrophage infiltration, inflammation, and fibrosis in CDMs. Thus, we combine chemical‐genetic profiling, mechanistic interrogation, and multicellular human cardiac tissue modelling to identify and pharmacologically interrogate a genotype‐associated ion‐mitochondrial vulnerability arising from reduced TET2 function in myeloid effector cells.

## 2 Results

### 2.1 Myeloid *TET2* Loss‐of‐Function Drives Cardiac Pathology in CDMs

Loss‐of‐function TET2 mutations in myeloid cells have been implicated in heart failure progression, but their direct effector‐cell consequences within human cardiac tissue remain incompletely understood [10, 23]. Because pathogenic TET2 driver mutations in clonal hematopoiesis commonly lead to reduced TET2 function, we used siRNA‐mediated TET2 depletion as a controlled functional surrogate to model the downstream consequence of TET2 loss‐of‐function in macrophages. This approach was not designed to reproduce the somatic DNA mutation event itself, or the stable hematopoietic stem‐cell fitness advantage and long‐term clonal evolution that define human CH. Rather, it allowed us to isolate macrophage‐intrinsic consequences of reduced TET2 function in an isogenic human cardiac co‐culture system.

THP‐1‐derived macrophages were transfected with scrambled siRNA (siScr) or TET2‐targeting siRNA (siTET2), followed by Qtracker labelling and co‐culture with CDMs (Figure 1A and Extended Data Figure S1A). Efficient TET2 depletion was confirmed at both the transcript and protein levels. In addition to reduced TET2 mRNA expression, Western blot analysis using antibodies against the C‐terminal and N‐terminal regions of TET2 showed that residual TET2 protein levels were reduced to 15.0% and 14.3% of siScr controls, respectively (Extended Data Figure S1B,C). We therefore refer to these cells as *TET2*‐knockdown macrophages throughout the manuscript and discuss the limitations of this acute knockdown model in relation to stable somatic TET2 mutations in human CH.

**FIGURE 1:**
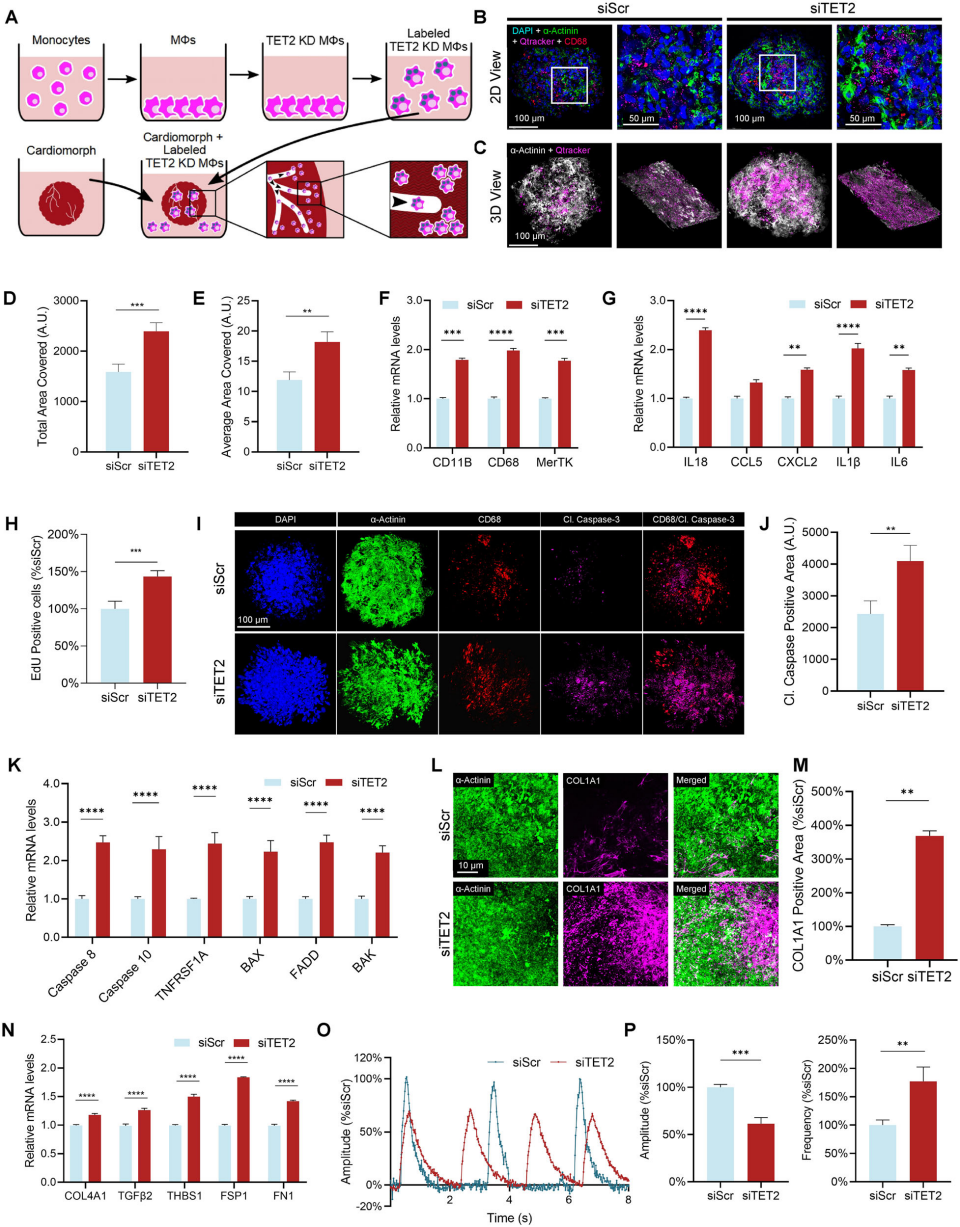
Acute *TET2*‐knockdown macrophage co‐culture modelling in CDMs reveals cardiac injury phenotypes. (A) Schematic of the TET2‐KD macrophage‐CDM co‐culture model. Monocytes were differentiated into macrophages (MΦs) using phorbol 12‐myristate 13‐acetate (PMA), transfected with scrambled siRNA (siScr) or siRNA targeting TET2 (siTET2), labelled with Qtracker, and co‐cultured with CDMs for 48 h to allow infiltration. (B) Two‐dimensional views of CDMs infiltrated by siScr or siTET2 macrophages. Nuclei were stained with DAPI (blue), cardiomyocytes with α‐actinin (green), exogenously added macrophages with Qtracker (magenta), and macrophages with CD68 (red). Scale bars, 100 µm; inset, 50 µm. (C) Three‐dimensional reconstructions of CDMs showing α‐actinin‐positive cardiomyocytes and Qtracker‐labelled macrophages. Scale bar, 100 µm. (D, E) Quantification of total (D) and average (E) macrophage‐infiltrated areas in CDMs as in (B). *n* = 3 biologically independent experiments per condition; more than 30 regions of interest from multiple organoids were analysed across the three replicates, with region‐level measurements averaged within each biological replicate to generate a single replicate‐level value before statistical analysis. (F) Relative mRNA levels of macrophage markers in CDMs infiltrated by siScr or siTET2 macrophages. *n* = 3 biologically independent replicates, 100 pooled CDMs per replicate. (G) Relative mRNA levels of inflammatory markers in CDMs infiltrated by siScr or siTET2 macrophages. *n* = 3 biologically independent replicates, 100 pooled CDMs per replicate. (H) Quantification of EdU (5‐Ethynyl‐2‐deoxyuridine) positive macrophages after siScr or siTET2 treatment. *n* = 4 biologically independent replicates. (I) Representative immunofluorescence 3D reconstructions of CDMs infiltrated by siScr or siTET2 macrophages, stained for nuclei (DAPI, blue), cardiomyocytes (α‐Actinin, green), macrophages (CD68, red), and apoptosis (cleaved‐Caspase‐3, magenta). The images are presented as a three‐dimensional (3D) reconstruction. Scale bar, 100 µm. (J) Quantification of cleaved‐Caspase‐3‐positive area within α‐actinin‐positive cardiomyocyte regions in CDMs as described in (I). *n* = 6 biologically independent samples per condition; for each biological replicate, three non‐overlapping cardiomorph areas were quantified and averaged to generate a single replicate‐level value. (K) Relative mRNA levels of apoptotic markers in CDMs infiltrated by siScr or siTET2 macrophages, *n* = 6 biologically independent replicates. (L) Representative immunofluorescence images of CDMs infiltrated by siScr or siTET2 macrophages, stained for cardiomyocytes (α‐Actinin, green) and fibrosis (COL1A1, magenta). Scale bar, 10 µm. (M) Quantification of COL1A1‐positive fibrotic area in CDMs as in (L). *n* = 6 biologically independent samples per condition. (N) Relative mRNA levels of fibrotic markers in CDMs infiltrated by siScr or siTET2 macrophages. *n* = 6 biologically independent replicates, 100 pooled CDMs per replicate. (O) Representative calcium flux transients in CDMs infiltrated by siScr or siTET2 macrophages. (P) Quantification of calcium‐transient amplitude and beating frequency in CDMs infiltrated by siScr or siTET2 macrophages, expressed relative to the siScr condition. *n* = 8 biologically independent replicates. Data are shown as mean ± SEM. Statistical significance for the quantitative comparisons in (D‐H, J, K, M, N and P) was assessed using two‐tailed unpaired t‐tests with Welch's correction. ^*^ *p* < 0.05, ^**^ *p* < 0.01, ^***^ *p* < 0.001, ^****^ *p* < 0.0001.

To verify that Qtracker‐positive infiltrating cells retained macrophage identity within CDMs, we further performed CD68 co‐immunostaining after 48 h of co‐culture. Representative confocal images showed extensive overlap between Qtracker and CD68 signals within infiltrating cells (Extended Data Figure S1D). Quantitative analysis confirmed that virtually all Qtracker‐positive cells were CD68‐positive (Extended Data Figure S1E), supporting the use of Qtracker signal to identify exogenously added macrophages within CDMs.

Qtracker labelling of exogenous macrophages together with CD68 immunostaining allowed us to evaluate macrophage infiltration into CDMs and distinguish exogenously added macrophages from endogenous CDM‐derived macrophages. As shown in Figure 1B–E, *TET2*‐KD macrophages infiltrated CDMs more extensively than controls, as supported by quantification using Z‐axis slices and 3D stack analyses (Extended Data Figure S1F,G). Elevated expression of the macrophage markers CD11B, CD68 and MerTK in *TET2*‐KD co‐cultures further supported the increased macrophage burden (Figure 1F). These results suggest that *TET2* loss‐of‐function enhances macrophage infiltration into cardiac tissue, a feature consistent with inflammatory tissue recruitment in CH‐associated disease. To explore downstream effects, we analyzed transcriptomic changes in CDMs co‐cultured with *TET2*‐KD macrophages. Inflammatory mediators, including interleukin‐1β (IL1B), interleukin‐6 (IL6), interleukin‐18 (IL18), and C‐X‐C motif chemokine ligand 2 (CXCL2), were significantly elevated in *TET2*‐KD conditions (Figure 1G), consistent with the pro‐inflammatory phenotype reported in clonal hematopoiesis [4, 10, 14, 24, 25]. EdU staining revealed increased proliferation of *TET2*‐KD macrophages compared to controls before co‐culture (Figure 1H and Extended Data Figure S1H). Together, these findings highlight a pro‐inflammatory and proliferative programme driven by *TET2*‐KD macrophages.

Given these inflammatory changes, we assessed cardiomyocyte apoptosis. Cleaved‐caspase‐3 (CASP3) staining confirmed increased apoptosis in CDMs exposed to TET2‐KD macrophages (Figure 1I,J), accompanied by elevated expression of pro‐apoptotic genes caspase 8 (CASP8) and 10 (CASP10), TNF Receptor Superfamily Member 1A (TNFRSF1A), BCL2‐associated X (BAX), BCL2 Antagonist/Killer 1 (BAK), and FAS‐associated death domain‐containing protein (FADD) (Figure 1K). These findings indicate that TET2 loss‐of‐function contributes to cardiomyocyte death, a component of cardiac dysfunction in CH‐associated disease. Next, we investigated fibrotic remodeling and contractile dysfunction, additional features of CH‐associated cardiac injury. TET2‐KD macrophage‐infiltrated CDMs exhibited significantly larger fibrotic areas (Figure 1L,M), with increased expression of pro‐fibrotic genes Transforming growth factor β2 (TGFB2), Type IV collagen alpha protein (COL4A1), Thrombospondin 1 (THBS1), Fibroblast specific protein 1 (FSP1), and Fibronectin 1 (FN1) (Figure 1N). CDMs infiltrated by TET2‐KD macrophages exhibited altered calcium‐handling dynamics, characterized by reduced calcium‐transient amplitude and increased beating frequency (Figure 1O,P). Together, these data indicate that CDMs capture key effector‐phase consequences of TET2 loss‐of‐function macrophage activity, including macrophage infiltration, inflammatory activation, cardiomyocyte apoptosis, fibrotic remodeling and impaired calcium‐handling dynamics. These findings support a direct contribution of *TET2*‐KD macrophages to cardiac‐tissue injury in this controlled human co‐culture system.

### 2.2 Chemical‐Genetic Profiling Identifies Ion Homeostasis as a Dominant Vulnerability in TET2‐Deficient Macrophages

Current therapies do not directly address the persistence of pathogenic *TET2*‐deficient myeloid effector cells, highlighting the need for targeted approaches. To identify compounds that preferentially compromise *TET2*‐KD macrophages, we performed chemical‐genetic screening using 489 cardiovascular disease‐related bioactive compounds from the TargetMol Anti‐Cardiovascular Disease Compound Library (Figure 2A,B and Extended Data Table S1). Inspection of top‐ranked hits highlighted several compounds linked to ion transport, mitochondrial stress, and stress‐signaling pathways, suggesting that disruption of intracellular ion homeostasis may represent a systems‐level vulnerability associated with TET2 deficiency. Following primary screening and secondary validation, eight compounds demonstrated preferential inhibition of *TET2*‐KD macrophage viability and proliferation with comparatively smaller effects on controls (Figure 2C,D). These findings indicate that TET2 deficiency may establish a broader vulnerability landscape characterized by sensitivity to perturbations in ion homeostasis and mitochondrial stress rather than a single compound‐specific pharmacologic sensitivity.

**FIGURE 2:**
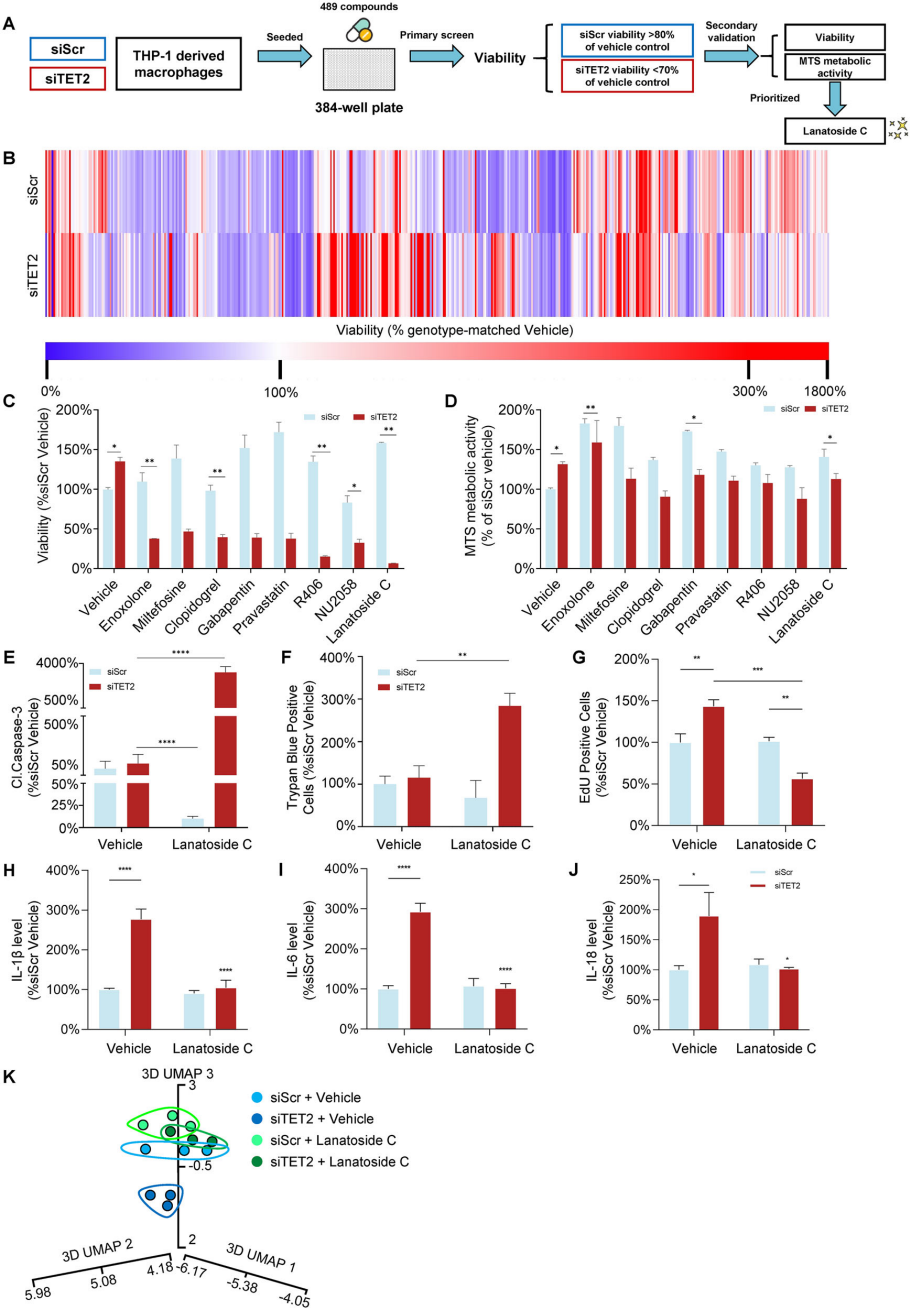
Chemical‐genetic screening identifies Lanatoside C as a selective inhibitor of *TET2*‐KD macrophages. (A) Screening workflow for identifying compounds preferentially targeting TET2‐KD macrophages. siScr and siTET2 THP‐1‐derived macrophages were plated in 384‐well format and treated with 489 compounds from the TargetMol Anti‐Cardiovascular Disease Compound Library for 48 h. Viability was measured by Alamar Blue and normalized to genotype‐matched DMSO controls. Hits were selected based on preservation of siScr viability > 80% and reduction of siTET2 viability < 70%, followed by secondary validation and prioritization of Lanatoside C. (B) Heatmap of relative cell viability in siScr or siTET2 macrophages treated with 489 cardiovascular disease‐related bioactive compounds (10 µM, 48 h). Viability was assessed by Alamar Blue and expressed relative to the corresponding genotype‐matched vehicle controls. *n* = 4 biologically independent replicates per treatment. (C) Quantification of cell viability by Alamar Blue assay in siScr or siTET2 macrophages treated with vehicle or a subset of 8 selected compounds. *n* = 8 biologically independent replicates per condition. (D) Quantification of proliferation‐associated metabolic activity using the MTS assay in siScr or siTET2 macrophages treated with vehicle or the same subset of 8 selected compounds. *n* = 4 biologically independent replicates per condition. (E) Quantification of cleaved‐Caspase‐3‐positive macrophages treated with siScr or siTET2, vehicle (DMSO), or Lanatoside C. *n* = 4 biologically independent replicates per treatment. (F) Quantification of cell viability by Trypan Blue exclusion in macrophages treated as in (E). *n* = 4 biologically independent replicates per condition. (G) Quantification of EdU‐positive macrophages treated with siScr or siTET2, vehicle, or Lanatoside C. *n* = 8 biologically independent replicates per condition. (H–J) Relative IL‐1β, IL‐6, and IL‐18 levels in macrophage monocultures following siScr or siTET2 transfection and treatment with vehicle or Lanatoside C. Cytokines were measured by ELISA and normalized to the siScr Vehicle group. Lanatoside C reduced the elevated inflammatory cytokine levels observed in TET2‐KD macrophages. *n* = 5 biologically independent replicates per condition. (K) UMAP plots from RNA sequencing of macrophages treated with siScr or siTET2 and vehicle or Lanatoside C. Each biological replicate comprised one million macrophages; *n* = 3 biologically independent replicates per condition. Data are shown as mean ± SEM. For the quantitative validation experiments in (C‐J), statistical significance was assessed using two‐way ANOVA followed by Tukey's multiple‐comparisons test, with the indicated pairwise comparisons shown in the figure. ^*^ *p* < 0.05, ^**^ *p* < 0.01, ^***^ *p* < 0.001, ^****^ *p* < 0.0001.

Lanatoside C, a cardiac glycoside that targets plasma membrane Na^+^/K^+^‐ATPase and has historically been used for cardiac indications, emerged as a top candidate for further mechanistic investigation [26, 27]. Given its reported pro‐apoptotic effects in other disease models, we further tested the effects of Lanatoside C on macrophage apoptosis and viability [28, 29]. Immunofluorescent confocal microscopy and cleaved‐caspase‐3 staining confirmed the pro‐apoptotic effects of Lanatoside C, while Trypan Blue staining demonstrated increased cell death in *TET2*‐KD macrophages (Figure 2E,F). Trypan Blue identifies non‐viable cells but does not distinguish apoptosis from necrosis; the concurrent increase in cleaved‐caspase‐3 supports apoptosis as a major component of the observed cell death in *TET2*‐KD macrophages.

To explore the molecular mechanism underlying the selectivity of Lanatoside C, transcriptomic analysis of *TET2*‐KD macrophages treated with Lanatoside C revealed upregulation of pro‐apoptotic genes and suppression of pro‐proliferative pathways (Extended Data Figure S2A,B). Consistent with these findings, EdU staining confirmed a significant reduction in *TET2*‐KD macrophage proliferation after Lanatoside C treatment (Figure 2G and Extended Data Figure S3). Given that inflammatory cytokines can influence macrophage survival and functional activation, we next examined whether Lanatoside C altered cytokine production in macrophage monocultures under the same treatment conditions. *TET2*‐KD macrophages exhibited markedly increased secretion of IL‐1β, IL‐6, and IL‐18 compared with siScr controls, whereas Lanatoside C reduced these cytokines to near‐baseline levels (Figure 2H–J). These data indicate that cytokine‐level changes accompany the response to Lanatoside C. However, together with the observed cleaved‐caspase‐3 activation, reduced viability, and impaired proliferation, these findings suggest that cytokine suppression is unlikely to solely account for the selective inhibition of *TET2*‐KD macrophages, which remains closely associated with apoptotic signaling. To visualize the transcriptomic shifts induced by Lanatoside C, we applied 3D Uniform Manifold Approximation and Projection (UMAP) [30, 31], which reduces high‐dimensional transcriptomic data into a 3D vector space for pattern recognition. In accordance with the transcriptomic profiles (Extended Data Figure S2A,B), UMAP showed that Lanatoside C shifted *TET2*‐KD macrophage profiles toward those of siScr controls (Figure 2K). Thus, these data identify Lanatoside C as a pharmacological tool for exploiting a TET2 loss‐of‐function‐associated vulnerability and motivates further mechanistic investigation of the underlying ion‐mitochondrial response.

### 2.3 Lanatoside C Selectively Triggers Apoptosis in *TET2*‐KD Macrophages Through Calcium‐Associated Mitochondrial Stress

Given prior evidence that TET2 deficiency enhances intracellular calcium signaling in macrophages, we next investigated whether dysregulated calcium homeostasis underlies the selective vulnerability of *TET2*‐KD macrophages to Lanatoside C (Figure 3A) [32]. Lanatoside C is a cardiac glycoside known to inhibit the Na^+^/K^+^‐ATPase, thereby indirectly modulating Na^+^/Ca^2+^ exchanger activity [33, 34, 35, 36, 37]. Transcript analysis showed reduced expression of genes encoding Na^+^/K^+^‐ATPase and Na^+^/Ca^2+^ exchanger components in Lanatoside C‐treated *TET2*‐KD macrophages (Figure 3B); these transcript‐level data do not directly establish transporter activity. This was accompanied by a marked increase in intracellular Cal‐520 Ca^2+^‐sensitive fluorescence following Lanatoside C treatment, which was significantly greater in *TET2*‐KD macrophages than in control cells (Figure 3C). To determine whether this calcium increase extended to mitochondria, we performed live‐cell Rhod‐2 imaging together with MitoTracker Green. *TET2*‐KD macrophages exhibited increased cellular Rhod‐2 fluorescence, which was further enhanced by Lanatoside C, with the strongest Rhod‐2 signal observed in Lanatoside C‐treated *TET2*‐KD macrophages (Extended Data Figure S4A,C). Consistently, Rhod‐2 fluorescence within MitoTracker Green‐positive regions was markedly increased in *TET2*‐KD macrophages following Lanatoside C treatment, indicating enhanced mitochondrial‐enriched Ca^2+^‐sensitive signal (Extended Data Figure S4B,C).

**FIGURE 3:**
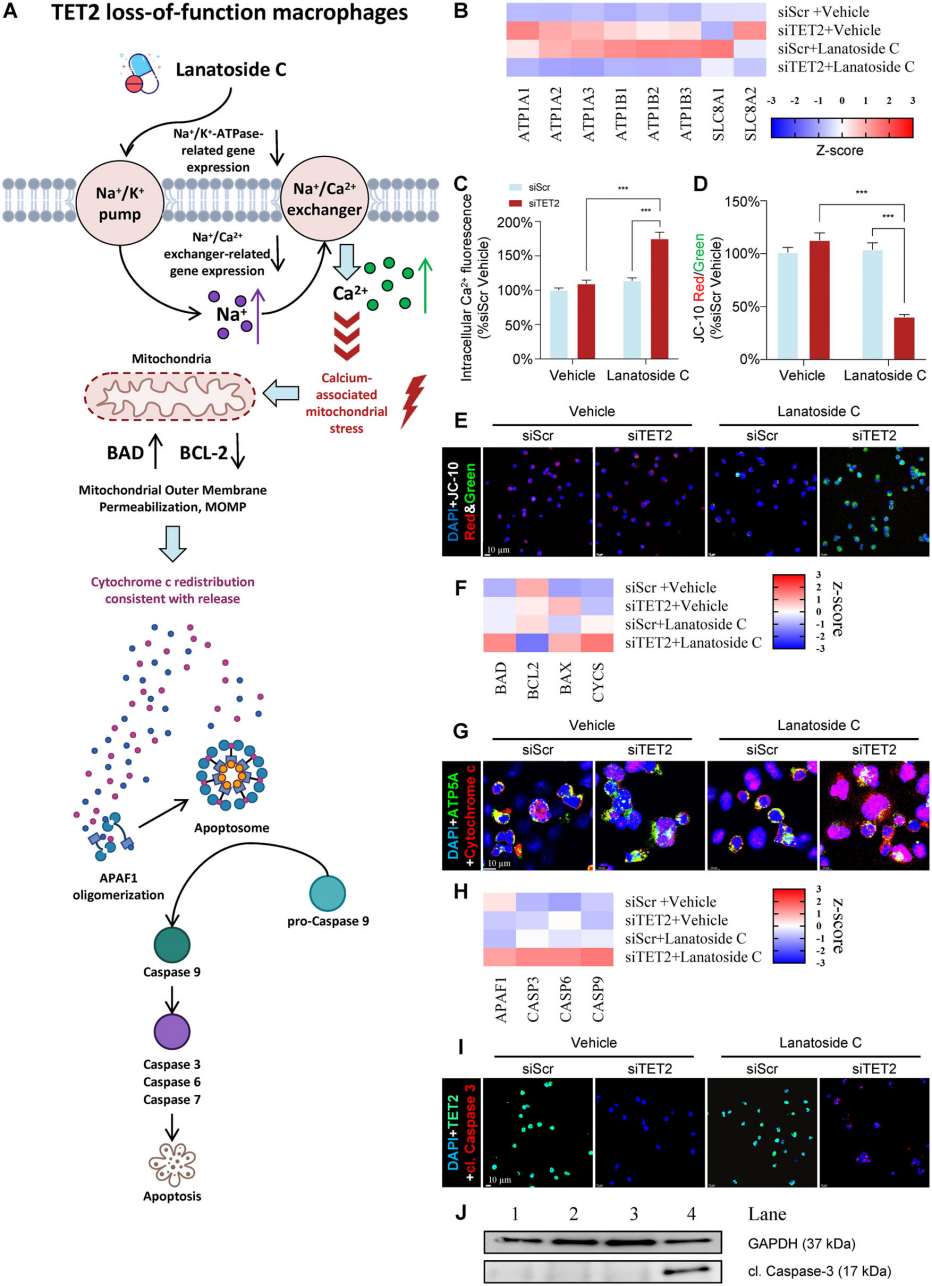
Lanatoside C selectively induces apoptosis in TET2‐knockdown macrophages through calcium‐associated mitochondrial stress. (A) Schematic representation of proposed pathways involved in Lanatoside C‐induced apoptosis in siTET2 macrophages. (B) Heatmap of Na^+^/K^+^ pump and Na^+^/Ca^2+^ exchanger gene expression (ATP1A1‐3, ATP1B1‐3, SLC8A1‐2) from bulk RNA sequencing of siScr or siTET2 macrophages treated with vehicle or Lanatoside C. *n* = 3 biologically independent replicates per condition. (C) Quantification of intracellular Ca^2+^‐sensitive Cal‐520 fluorescence in siScr and siTET2 macrophages treated with vehicle or Lanatoside C. Fluorescence values were normalized to the siScr vehicle group. *n* = 8 biologically independent replicates per condition. (D) Quantification of JC‐10 red/green fluorescence ratio in siScr and siTET2 macrophages treated with vehicle or Lanatoside C. *n* = 8 biologically independent replicates per condition. (E) Representative JC‐10 fluorescence images of siScr and siTET2 macrophages treated with vehicle or Lanatoside C, corresponding to the quantification shown in (D). Red fluorescence indicates JC‐10 J‐aggregates in polarized mitochondria, whereas green fluorescence indicates JC‐10 monomers in depolarized mitochondria. Scale bar, 10 µm. (F) Heatmap of mitochondrial depolarization‐related gene expression from bulk RNA sequencing of siScr or siTET2 macrophages treated with vehicle or Lanatoside C. *n* = 3 biologically independent replicates per condition. (G) Representative immunofluorescence images of siScr and siTET2 macrophages stained for ATP5A (green) and Cytochrome c (red) after treatment with vehicle or Lanatoside C. Scale bar, 10 µm. (H) Heatmap of apoptosis‐related gene expression from bulk RNA sequencing of siScr or siTET2 macrophages treated with vehicle or Lanatoside C. *n* = 3 biologically independent replicates per condition. (I) Representative immunofluorescence images of siScr and siTET2 macrophages treated with vehicle or Lanatoside C and co‐stained for TET2 (green), cleaved‐Caspase‐3 (red), and DAPI (blue). Scale bar, 10 µm. (J) Representative Western blot showing cleaved Caspase‐3 protein abundance in siScr and siTET2 macrophages after treatment with vehicle or Lanatoside C. GAPDH served as loading control. Lanes 1–4 represent siScr + vehicle, siTET2 + vehicle, siScr + Lanatoside C, and siTET2 + Lanatoside C, respectively. Data are shown as mean ± SEM. Expression values in the heatmaps are shown as Z‐scores. For the quantitative comparisons in (C,D), statistical significance was assessed using two‐way ANOVA followed by Tukey's multiple‐comparisons test, with the indicated pairwise comparisons shown in the figure. ^*^ *p* < 0.05, ^**^ *p* < 0.01, ^***^ *p* < 0.001, ^****^ *p* < 0.0001.

Excess intracellular calcium and enhanced mitochondrial‐enriched Ca^2+^ signals can promote mitochondrial dysfunction and intrinsic apoptotic signaling [38, 39, 40, 41]. Consistent with this, assessment of mitochondrial membrane potential using JC‐10 demonstrated pronounced mitochondrial depolarization in Lanatoside C‐treated *TET2*‐KD macrophages, whereas control macrophages showed comparatively limited changes (Figure 3D,E and Extended Data Figure S5). At the transcriptional level, Lanatoside C shifted the balance of BCL‐2 family regulators in *TET2*‐KD macrophages toward a pro‐apoptotic state, with increased expression of pro‐apoptotic regulators including BAD and BAX and reduced expression of anti‐apoptotic BCL2 (Figure 3F), consistent with increased susceptibility to mitochondrial outer membrane permeabilization (MOMP).

Consistent with this mitochondrial stress response, immunofluorescence analysis showed redistribution of cytochrome c away from ATP5A‐positive mitochondrial regions in Lanatoside C‐treated *TET2*‐KD macrophages, while immunoblotting further demonstrated altered cytochrome c abundance under the same conditions (Figure 3G and Extended Data Figure S6A–D). These findings are consistent with cytochrome c redistribution during mitochondrial apoptotic signaling [38, 39, 40, 41]. Downstream apoptotic signaling was further supported by increased expression of CASP9, CASP3 and CASP6, together with robust activation of cleaved Caspase‐3 (Figure 3H–J) [42, 43, 44]. *TET2*‐matched immunofluorescence confirmed that cleaved Caspase‐3 induction occurred predominantly in siTET2 macrophages following Lanatoside C treatment, whereas siScr macrophages with preserved TET2 expression remained comparatively resistant under the same conditions (Figure 3I,J and Extended Data Figure S7A,B).

Together, these findings indicate that *TET2*‐KD macrophages occupy a calcium‐sensitized ion‐mitochondrial state that is tolerated at baseline but is selectively pushed by Lanatoside C toward increased mitochondrial‐enriched Ca^2+^‐sensitive signal, mitochondrial depolarization and intrinsic apoptotic signaling, whereas control macrophages remain largely resistant under the same conditions. These results prompted us to test whether preferential reduction of *TET2*‐KD macrophages is sufficient to suppress inflammation and improve tissue‐level homeostasis in a human cardiac context.

### 2.4 Lanatoside C Attenuates Inflammatory and Cardiac Injury Phenotypes in TET2‐KD Macrophage‐Infiltrated CDMs

Having established that Lanatoside C preferentially induces calcium‐associated mitochondrial stress and apoptosis in *TET2*‐KD macrophages, we next examined whether reducing the burden of these macrophages ameliorates cardiac‐tissue phenotypes in the 3D cardiomorph co‐culture system. Control or *TET2*‐KD macrophages were Qtracker‐labelled and co‐cultured with CDMs for 48 h in the presence of vehicle or Lanatoside C. Consistent with the preferential apoptotic response observed in macrophage monoculture, Lanatoside C markedly reduced the persistence of *TET2*‐KD macrophages within CDMs while exerting comparatively limited effects on control macrophages (Extended Data Figure S8A–C).

Because infiltrating *TET2*‐KD macrophages generate a pro‐inflammatory milieu, we next assessed inflammatory cytokine production in CDM co‐cultures. CDMs exposed to vehicle‐treated *TET2*‐KD macrophages exhibited significantly elevated IL‐1β, IL‐6 and IL‐18 compared with control co‐cultures. Lanatoside C reduced these cytokine concentrations toward baseline levels (Figure 4A), consistent with suppression of macrophage‐associated inflammatory signaling. Immunofluorescence analysis further revealed increased cardiomyocyte apoptosis in CDMs infiltrated by *TET2*‐KD macrophages, as indicated by elevated cleaved Caspase‐3 staining in α‐actinin‐positive cardiomyocytes. Lanatoside C significantly reduced this apoptotic signal (Figure 4B–D). In parallel, cardiac troponin T release into the culture medium was increased in CDMs exposed to *TET2*‐KD macrophages and was significantly reduced following Lanatoside C treatment (Figure 4E), consistent with reduced cardiomyocyte injury.

**FIGURE 4:**
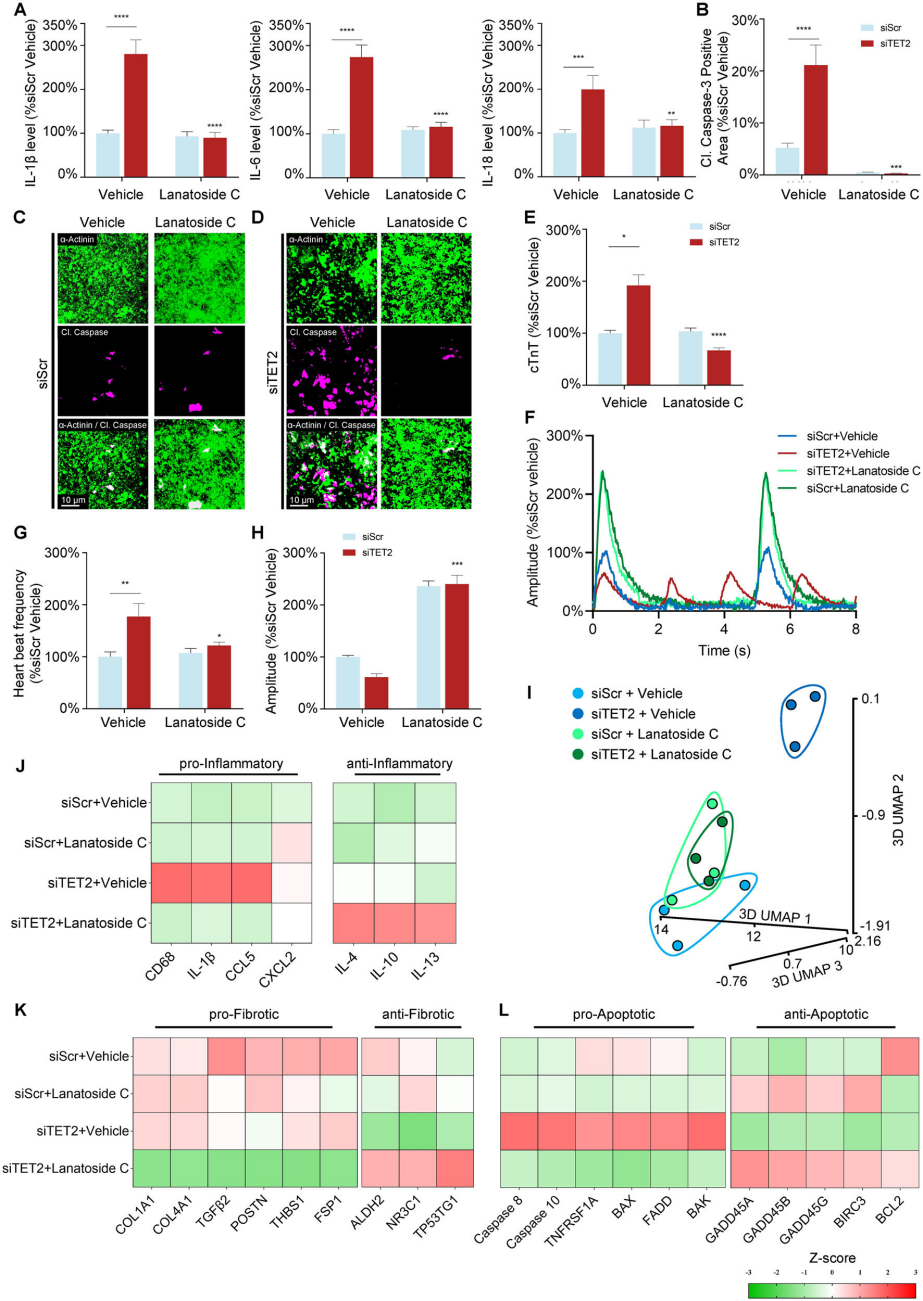
Lanatoside C attenuates cardiac injury phenotypes in *TET2*‐KD macrophage‐infiltrated CDMs. (A) Relative IL‐1β, IL‐6, and IL‐18 levels in the culture medium of CDMs infiltrated by siScr or siTET2 macrophages and treated with vehicle or Lanatoside C (10 µM, 48 h), measured by ELISA and normalized to the siScr Vehicle group. *n* = 8 biologically independent replicates per condition. (B) Quantification of cleaved Caspase‐3‐positive areas in CDMs infiltrated by siScr or siTET2 macrophages treated with vehicle or Lanatoside C. *n* = 8 biologically independent samples per condition. (C,D) Representative immunofluorescence images of CDMs stained with α‐actinin (green) and cleaved‐Caspase‐3 (magenta) after treatment as in (B). Scale bar, 10 µm. (E) Quantification of cardiac troponin T (cTnT) release into culture media of CDMs infiltrated by siScr or siTET2 macrophages treated with vehicle or Lanatoside C. *n* = 4 biologically independent replicates per condition. (F) Representative calcium flux transients in CDMs infiltrated by siScr or siTET2 macrophages treated with vehicle or Lanatoside C. (G,H) Quantification of beating frequency (G) and amplitude (H) in CDMs infiltrated by siScr or siTET2 macrophages treated with vehicle or Lanatoside C. *n* = 8 biologically independent replicates per condition. (I) UMAP plots from RNA sequencing of CDMs infiltrated by siScr or siTET2 macrophages treated with vehicle or Lanatoside C. *n* = 3 biologically independent replicates per condition, each with 100 pooled CDMs. (J–L) Heatmaps of inflammatory (J), fibrotic (K), and apoptotic (L) marker expression from bulk RNA sequencing of CDMs infiltrated by siScr or siTET2 macrophages treated with vehicle or Lanatoside C. *n* = 3 biologically independent replicates per condition. Data are shown as mean ± SEM. Expression values in the heatmaps are shown as Z‐scores. For the quantitative comparisons in (A,B,E,G,H), statistical significance was assessed using two‐way ANOVA followed by Tukey's multiple‐comparisons test, with the indicated pairwise comparisons shown in the figure. ^*^ *p* < 0.05, ^**^ *p* < 0.01, ^***^ *p* < 0.001, ^****^ *p* < 0.0001.

We next examined fibrotic remodeling. CDMs infiltrated by TET2‐KD macrophages displayed increased COL1A1 deposition compared with control co‐cultures, whereas Lanatoside C substantially reduced COL1A1 fluorescence intensity (Extended Data Figure S9A,B). Given the combined suppression of inflammation, apoptosis and fibrosis, we next assessed the effects of Lanatoside C on cardiomorph calcium‐handling dynamics. *TET2*‐KD macrophage‐infiltrated CDMs exhibited reduced calcium‐transient amplitude and increased beating frequency compared with control co‐cultures (Figure 4F–H). Lanatoside C increased the depressed calcium‐transient amplitude and partially normalized the elevated beating frequency in *TET2*‐KD macrophage‐infiltrated CDMs. Notably, Lanatoside C also increased calcium‐transient amplitude in control CDMs, consistent with the known direct inotropic actions of cardiac glycosides; therefore, the functional effects observed in the co‐culture system cannot be attributed exclusively to macrophage elimination.

Transcriptomic profiling supported these phenotypic observations. Bulk RNA sequencing and qPCR analyses showed lower expression of pro‐inflammatory genes (CD68, IL1B, CCL5, CXCL2) and pro‐fibrotic markers (COL1A1, COL4A1, TGFB2, POSTN, THBS1), and higher expression of anti‐inflammatory (IL4, IL10, IL13) and cytoprotective or anti‐fibrotic genes (ALDH2, NR3C1, TP53TG1) (Figure 4J–L; Extended Data Figures S10 and S11). In parallel, expression of pro‐apoptotic genes (CASP8, CASP10, BAX, FADD) was reduced, whereas anti‐apoptotic regulators (BCL2, GADD45A/B/G, BIRC3) were increased, resulting in an elevated BCL2/BAX ratio (Extended Data Figure S10V). Finally, unsupervised dimensionality reduction using UMAP showed that Lanatoside C‐treated *TET2*‐KD macrophage‐infiltrated CDMs shifted toward the transcriptional state of control co‐cultures (Figure 4I). Together, these findings indicate that reduction of *TET2*‐KD macrophage‐associated inflammatory and tissue‐remodelling phenotypes is accompanied by improved calcium‐handling dynamics, while also highlighting a direct pharmacological effect of Lanatoside C on cardiac tissue.

### 2.5 Lanatoside C Attenuates Tet2‐Deficient Donor‐Derived Myeloid Representation in a Partial Donor‐Cell Reconstitution Model

To determine whether the preferential effects of Lanatoside C on *TET2*‐deficient myeloid cells extend to an in vivo setting, we evaluated its activity in a partial donor‐cell reconstitution model using separate Tet2 donor‐genotype cohorts. Bone marrow cells from either Tet2^−/−^ or Tet2^+/+^ CD45.2^+^ donor mice were transplanted separately into irradiated CD45.1^+^ recipient mice, allowing CD45.2^+^ donor‐derived cells to be distinguished from CD45.1^+^ recipient‐derived cells by flow cytometry (Figure 5A). The donor inocula contained only CD45.2+ donor marrow; CD45.1+ leukocytes detected after transplantation were therefore recipient‐derived. Each recipient received 5 × 10^6^ donor bone marrow cells intravenously per day for three consecutive days. Four weeks after transplantation, before treatment initiation, peripheral blood was collected to assess baseline donor‐derived CD45.2^+^ leukocyte chimerism. Mice subsequently received vehicle or Lanatoside C for one month, with endpoint samples collected approximately eight weeks after transplantation (week 8).

**FIGURE 5:**
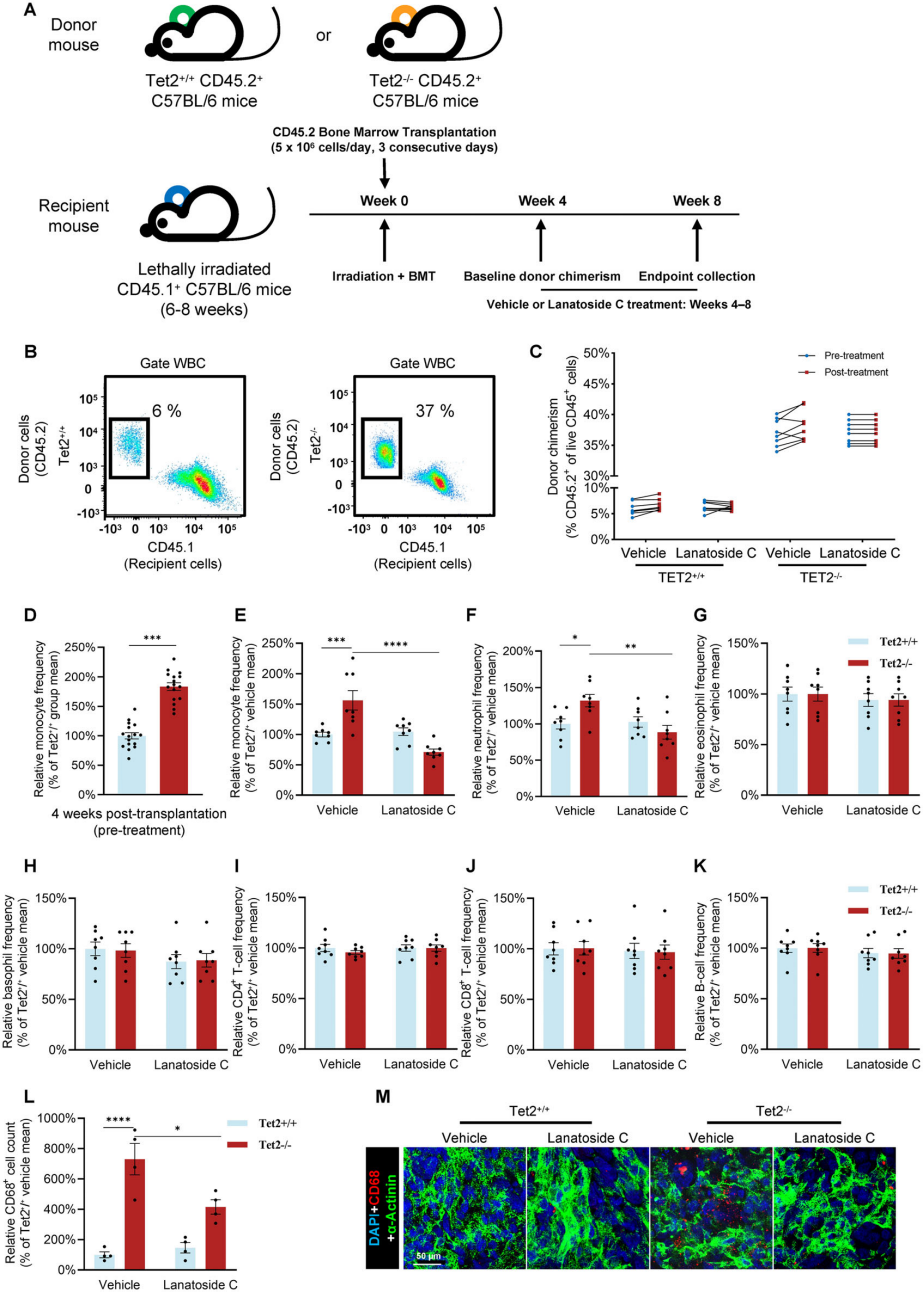
Lanatoside C attenuates Tet2‐deficient donor‐derived myeloid representation in a partial donor‐cell reconstitution model. (A) Schematic of the bone marrow transplantation strategy. Bone marrow cells from either Tet2^+/+^ or Tet2^−/−^ CD45.2^+^ donor mice were transplanted separately into irradiated CD45.1^+^ recipient mice. Conditioning consisted of 11 Gy total‐body irradiation in two 5.5‐Gy fractions separated by 4 h. Each recipient received 5 × 10^6^ donor cells intravenously per day for three consecutive days. Week 0 denotes irradiation and initiation of the three‐day transplantation schedule. At week 4, baseline peripheral‐blood donor chimerism was assessed before treatment allocation. Vehicle or Lanatoside C was then administered daily for one month (approximately weeks 4–8), with endpoint peripheral‐blood and cardiac‐tissue collection at approximately week 8. The experiment achieved partial donor reconstitution, consistent with incomplete marrow ablation inferred from blood chimerism. (B) Representative flow‐cytometry plots showing CD45.2^+^ donor‐derived leukocytes among circulating live CD45^+^ white blood cells at four weeks after transplantation, before treatment initiation, in Tet2^+/+^ and Tet2^−/−^ donor‐cell recipients. CD45.1+ events identify recipient‐derived leukocytes; these measurements show partial peripheral‐blood donor chimerism, not bone‐marrow chimerism. The percentages shown represent individual illustrative mice rather than cohort means. **(C)** Total circulating donor‐cell chimerism before and after the one‐month treatment period, quantified as the percentage of CD45.2^+^ donor‐derived cells among total live circulating CD45^+^ leukocytes. Paired points joined by a line represent the same recipient at week 4 (pre‐treatment) and approximately week 8 (post‐treatment). *n* = 8 mice per treatment group. The interrupted y‐axis separates the low and high donor‐chimerism ranges. (D) Baseline monocyte frequency within circulating live CD45.2^+^ donor‐derived leukocytes at four weeks after transplantation, before treatment allocation. Each dot represents one independent recipient mouse (*n* = 16 per donor‐genotype cohort, comprising the mice subsequently assigned to the two treatment groups). Each mouse‐level monocyte frequency was divided by the mean frequency of the Tet2^+/+^ donor‐cell cohort and multiplied by 100. The reference‐group mean, rather than each individual control value, was set to 100%. (E–K) Endpoint frequencies of monocytes (E), neutrophils (F), eosinophils (G), basophils (H), CD4^+^ T cells (I), CD8^+^ T cells (J), and B cells (K) within circulating live CD45.2^+^ donor‐derived leukocytes after one month of vehicle or Lanatoside C treatment (approximately eight weeks after transplantation). Each dot represents one independent recipient mouse (*n* = 8 per treatment group). For each lineage, the individual‐mouse frequency was divided by the mean frequency of the Tet2^+/+^ vehicle group and multiplied by 100. A plotted value of 200% therefore denotes twice the reference‐group mean lineage frequency. These are normalized per‐mouse measurements, not unnormalized lineage percentages, total donor chimerism or absolute blood‐cell counts. (L) Quantification of CD68^+^ macrophage accumulation in cardiac tissue from Tet2^+/+^ or Tet2^−/−^ donor‐cell recipient mice treated with vehicle or Lanatoside C. CD68^+^ macrophage numbers were normalized to the Tet2^+/+^ Vehicle group, which was set to 100%. *n* = 4 mice per group; each data point represents the mean value from one mouse calculated from 3 non‐overlapping cardiac fields. (M) Representative confocal whole‐mount immunofluorescence images of mouse ventricular myocardium showing sarcomeric α‐actinin‐positive cardiomyocytes (green), CD68‐positive macrophages (red), and DAPI‐labelled nuclei (blue). A single optical section is shown. CD68 does not distinguish donor‐derived from recipient‐derived or resident macrophages. Scale bar, 50 µm. Data are presented as mean ± SEM. *n* = 16 mice per donor‐genotype cohort for the baseline analysis in (D), *n* = 8 mice per treatment group for the peripheral‐blood analyses in (C, E–K), and *n* = 4 mice per group for the cardiac CD68 analysis in (L). For the longitudinal donor‐chimerism analysis in (C), pre‐ and post‐treatment measurements from the same mouse were analyzed using two‐way repeated‐measures ANOVA followed by Šídák's multiple‐comparisons test. Statistical significance in (D) was assessed using a two‐tailed unpaired t‐test with Welch's correction. For (E‐L), statistical significance was assessed using two‐way ANOVA followed by Tukey's multiple‐comparisons test, with the indicated pairwise comparisons shown in the figure. ^*^ *p* < 0.05, ^**^ *p* < 0.01, ^***^ *p* < 0.001, ^****^ *p* < 0.0001.

Flow cytometry demonstrated partial peripheral‐blood donor chimerism rather than complete donor replacement. At four weeks, the representative plots in Figure 5B show CD45.2^+^ donor‐derived cells comprising approximately 6% and 37% of live circulating CD45+ leukocytes in the illustrated Tet2^+/+^ and Tet2^−/−^ donor‐cell recipients, respectively. These percentages are individual examples, not cohort means; individual‐mouse chimerism measurements are shown in Figure 5C. The remaining CD45.1^+^ leukocytes were recipient‐derived. The substantial recipient‐derived contribution is consistent with incomplete marrow ablation and partial donor reconstitution. This interpretation is inferred from peripheral‐blood chimerism: marrow ablation, bone‐marrow chimerism and recipient progenitors were not directly assessed. The data therefore cannot distinguish persistence of radio‐resistant mature cells from recovery by residual recipient progenitors. The separate donor‐genotype cohorts were not a mixed‐donor competitive transplantation experiment, and these blood measurements do not establish a Tet2‐associated competitive stem‐cell fitness advantage.

Total donor chimerism was reassessed after the one‐month treatment period using the same CD45.1/CD45.2 gating strategy, with week‐4 and approximately week‐8 measurements paired within individual recipient mice (*n* = 8 per treatment group; Figure 5C). No significant pre‐to‐post‐treatment change in total CD45.2+ donor chimerism was detected in Tet2^+/+^ vehicle‐treated, Tet2^+/+^ Lanatoside C‐treated, Tet2^−/−^ vehicle‐treated, or Tet2^−/−^ Lanatoside C‐treated recipients. Thus, the subsequent lineage‐level effects occurred without a detectable change in overall circulating donor‐cell chimerism during the treatment period. The absence of a statistically significant change does not establish equivalence. These two time‐point measurements, however, do not define clone‐growth kinetics or hematopoietic stem‐cell fitness.

Detailed lineage analysis of the pre‐treatment CD45.2^+^ donor‐derived compartment further showed that Tet2^−/−^ donor‐cell recipients exhibited increased circulating monocyte representation relative to Tet2^+/+^ donor‐cell recipients, with each mouse‐level monocyte frequency normalized to the mean of the Tet2^+/+^ donor‐cell cohort (*n* = 16 mice per donor‐genotype cohort before treatment allocation; Figure 5D). After baseline chimerism assessment, recipient mice were randomized to receive daily intraperitoneal injections of vehicle or Lanatoside C (5 mg/kg/day) for one month. At the endpoint, approximately eight weeks after transplantation, peripheral‐blood lineage composition was quantified within the live CD45.2^+^ donor‐derived leukocyte compartment. Figure 5E–K shows individual‐mouse values (*n* = 8 per treatment group), normalized for each lineage to the Tet2^+/+^ vehicle‐group mean. Thus, 200% denotes twice the reference‐group mean lineage frequency, not 200% of the circulating cells; individual control values were not each fixed at 100%. Lanatoside C significantly reduced the relative frequency of donor‐derived monocytes in Tet2^−/−^ donor‐cell recipients compared with vehicle‐treated Tet2^−/−^ animals, whereas no comparable reduction was observed in Tet2^+/+^ donor‐cell recipients (Figure 5E). These comparisons concern monocyte frequencies within the donor‐derived compartment, not absolute circulating monocyte counts. The treatment comparison within each donor‐genotype cohort is distinct from the between‐genotype difference in baseline donor chimerism.

Within the same circulating CD45.2^+^ donor‐derived compartment, neutrophil frequencies were elevated in vehicle‐treated Tet2^−/−^ donor‐cell recipients relative to Tet2^+/+^ controls. Lanatoside C treatment reduced donor‐derived neutrophil representation in Tet2^−/−^ donor‐cell recipients toward the range observed in Tet2^+/+^ animals (Figure 5F). By contrast, eosinophil and basophil populations were not significantly altered by Tet2 genotype or Lanatoside C treatment (Figure 5G,H). Within the measured donor‐derived compartment, Lanatoside C did not comparably reduce the assessed lymphoid populations. Frequencies of CD4^+^ T cells, CD8^+^ T cells and B cells within the circulating CD45.2^+^ donor‐derived compartment were not significantly different between vehicle‐ and Lanatoside C‐treated mice in both Tet2^+/+^ and Tet2^−/−^ donor‐cell recipient groups (Figure 5I–K). Together, the within‐cohort comparisons show reduced donor‐derived monocyte and neutrophil frequencies in Tet2‐deficient donor‐cell recipients, without corresponding detected reductions in the measured donor‐derived lymphoid frequencies. These normalized frequencies describe donor‐compartment composition, not absolute lineage counts or total mutant‐cell burden.

To determine whether circulating myeloid changes were accompanied by cardiac tissue changes, we performed whole‐mount immunofluorescence staining of ventricular myocardium for CD68 and sarcomeric α‐actinin in the available transplantation cohort. Tet2^−/−^ donor‐cell recipients showed increased cardiac CD68^+^ macrophage accumulation compared with Tet2^+/+^ controls, whereas Lanatoside C reduced the CD68^+^ macrophage burden in Tet2^−/−^ recipient hearts (Figure 5L,M). Confocal imaging localized CD68^+^ macrophages within α‐actinin‐positive ventricular myocardium. Because CD68 staining alone does not distinguish donor‐derived Tet2‐deficient macrophages from resident or recipient‐derived macrophages, these data indicate reduced total cardiac macrophage accumulation rather than definitive suppression of donor‐derived macrophage infiltration.

Together, these findings provide complementary in vivo evidence that Lanatoside C alters the relative representation of *Tet2*‐deficient donor‐derived monocytes and neutrophils under the tested conditions. They do not demonstrate wholesale depletion of Tet2‐deficient hematopoiesis or establish that apoptosis accounts for the lineage changes in vivo. Direct quantification of donor‐derived macrophage infiltration into cardiac tissue was not possible in this cohort. Competitive transplantation, longitudinal clonal tracking, absolute blood‐cell counts and lineage‐resolved cardiac profiling will be required to determine effects on stem‐cell fitness, clonal architecture and tissue infiltration.

All 32 recipient mice completed the study without mortality or overt treatment‐related adverse events. Complete blood counts, hemoglobin concentrations, erythroid indices and absolute blood‐cell counts were not systematically measured. Survival and relative lineage frequencies therefore cannot exclude subclinical anemia or other cytopenias, and this experiment does not establish hematological safety.

### 2.6 Reduced TET2 Function Sensitizes Human Macrophages to Lanatoside C

We next examined whether the differential Lanatoside C sensitivity observed in the THP‐1‐derived macrophage model could be reproduced in primary human macrophages. CD14^+^ monocytes from four independent human donors were differentiated into macrophages and transfected with siScr or siTET2 before exposure to a concentration series of Lanatoside C. siTET2‐transfected primary macrophages showed a greater concentration‐dependent loss of viability and induction of apoptosis than matched siScr‐transfected controls (Extended Data Figure S12A,B). Four‐parameter logistic modelling of these complementary readouts further confirmed the differential Lanatoside C sensitivity associated with experimental reduction of TET2 in primary human macrophages (Extended Data Figure S12C).

We next asked whether this differential sensitivity was retained in patient‐derived macrophages carrying endogenous TET2 mutations, without experimental TET2 silencing. Across eight independent individuals per group, TET2‐mutant macrophages showed a left‐shifted concentration‐response to Lanatoside C relative to the *TET2*‐WT comparator group (Extended Data Figure S12D). Four‐parameter logistic modelling yielded IC_50_ point estimates of 0.138 µM for *TET2*‐mutant macrophages and 3.048 µM for *TET2*‐WT macrophages. The TET2‐mutant estimate was well bounded within the concentration range tested (95% CI, 0.106‐0.177 µM), whereas the upper confidence limit for the *TET2*‐WT estimate could not be determined. The apparent greater‐than‐20‐fold separation between the point estimates should therefore be interpreted cautiously (Extended Data Figure S12E).

The estimated IC_50_ of 0.138 µM in TET2‐mutant patient‐derived macrophages was numerically within the early post‐dose serum concentrations reported in a historical human intravenous study of Lanatoside C. In that study, administration of approximately 1.6 mg Lanatoside C intravenously resulted in mean serum concentrations of 0.25 µg mL^−1^ immediately after dosing and 0.10 µg mL^−1^ at 7.5 min, corresponding to approximately 0.254 and 0.102 µM, respectively [45, 46, 47]. By contrast, the estimated IC50 for TET2‐WT macrophages was 3.048 µM. This historical comparison is provided for pharmacological context only and does not establish a clinically usable therapeutic window.

## 3 Discussion

Here we identify TET2 loss‐of‐function‐associated disruption of ion‐mitochondrial homeostasis as a pharmacologically exploitable vulnerability in myeloid effector cells. Although TET2 loss‐of‐function mutations are strongly associated with cardiovascular inflammation and adverse cardiac remodeling [1, 48, 49, 50], therapeutic strategies have primarily focused on downstream cytokine pathways rather than directly targeting pathogenic *TET2*‐deficient myeloid effector cells [51, 52]. We show that reduced TET2 function establishes a calcium‐sensitized ion‐mitochondrial state that confers preferential susceptibility to further perturbation of intracellular ion balance. Pharmacological exploitation of this vulnerability promotes preferential apoptosis of *TET2*‐deficient macrophages, suppresses inflammatory and tissue‐remodeling phenotypes in multicellular human cardiac organoids, and attenuates *Tet2*‐deficient myeloid representation in vivo.

The mechanistic advance is the identification of a calcium‐associated mitochondrial stress state accompanying preferential Lanatoside C sensitivity in *TET2*‐deficient macrophages. Consistent with the known ion‐handling effects of cardiac glycosides, treatment increased intracellular calcium‐sensitive fluorescence and mitochondrial‐enriched Rhod‐ 2 signals together with mitochondrial depolarization and intrinsic apoptotic signaling. Control macrophages were comparatively resistant under the same conditions. The concordance of these measurements supports a pharmacologically exploitable, TET2‐associated vulnerability, but the endpoint experiments do not resolve the temporal order of calcium dysregulation and apoptosis or establish calcium dysregulation as necessary for cell death.

A central question is whether preferential suppression of pathogenic *TET2*‐deficient myeloid effector cells is sufficient to attenuate cardiac injury phenotypes. Using a multicellular human cardiomorph platform, we show that *TET2*‐KD macrophages drive a coordinated programme of inflammatory cytokine release, cardiomyocyte apoptosis, fibrotic remodeling and abnormal calcium‐handling dynamics. Lanatoside C reduced *TET2*‐KD macrophage burden and was accompanied by suppression of inflammatory signaling, reduced cardiomyocyte injury and fibrosis, and improvement of abnormal calcium‐handling phenotypes. Notably, Lanatoside C also increased calcium‐transient amplitude in control CDMs, consistent with the direct inotropic actions of cardiac glycosides. The functional effects observed in the cardiomorph system therefore cannot be attributed exclusively to macrophage elimination. Nevertheless, the preferential sensitivity of *TET2*‐KD macrophages, together with the reduction in macrophage burden and associated inflammatory and tissue‐remodeling phenotypes, supports a substantial contribution of *TET2*‐deficient macrophage activity to cardiac‐tissue injury in this model.

The cardiomorph platform is integral to this conclusion. By recapitulating key cellular, structural and functional features of human cardiac tissue, this system enables controlled dissection of immune‐cardiac interactions that cannot be resolved using reductionist monoculture systems or inferred from murine models alone. Although no in vitro model fully captures systemic influences such as neurohumoral regulation or ageing, the convergence of cardiomorph, primary‐human‐cell and mouse data supports the broader relevance of a *TET2* loss‐of‐function‐associated myeloid vulnerability. The mouse findings provide a narrower, complementary observation: *in* a partial donor‐cell reconstitution model, Lanatoside C reduced relative *Tet2*‐deficient donor‐derived myeloid representation without a detected reduction in total donor chimerism or comparable reductions in the measured donor‐derived lymphoid frequencies. Substantial recipient‐derived leukocyte representation is consistent with incomplete marrow ablation and partial donor reconstitution, inferred from blood measurements rather than direct examination of the marrow. Consequently, this experiment does not establish complete clone depletion, normal absolute blood‐cell counts or altered hematopoietic stem‐cell fitness. The human‐cell evidence for differential drug sensitivity does not depend on assuming near‐complete donor replacement in mice.

Several limitations should be considered. First, the human co‐culture system models acute TET2 loss‐of‐function through siRNA‐mediated knockdown in THP‐1‐derived macrophages and therefore does not reproduce the stable somatic mutation, hematopoietic stem‐cell origin, persistent epigenetic remodeling or long‐term clonal competition that define human *TET2*‐mutant clonal hematopoiesis. Our findings consequently address the vulnerability and pathogenic activity of downstream *TET2*‐deficient myeloid effector cells rather than the establishment, maintenance or evolution of the mutant clone. Recent work has further demonstrated that inherited and acquired regulators of hematopoietic stem‐cell fitness can critically shape clonal expansion and long‐term dominance [53]. Competitive transplantation, longitudinal variant‐allele‐frequency tracking and bone‐marrow hematopoietic stem and progenitor cell profiling will therefore be required to determine whether pharmacological targeting of this vulnerability alters mutant stem‐cell fitness or long‐term clonal behavior. Although Rhod‐2 imaging together with MitoTracker Green provides spatially resolved evidence of enhanced mitochondrial‐enriched Ca^2+^‐sensitive fluorescence, these measurements do not provide absolute mitochondrial Ca^2+^ concentrations or resolve mitochondrial Ca^2+^ flux kinetics. Future studies using genetically encoded mitochondrial Ca^2+^ reporters would enable higher‐resolution analysis of these dynamics. In addition, calcium chelation or targeted perturbation of mitochondrial Ca^2+^ uptake and Na^+^/Ca^2+^ exchange will be required to establish whether calcium dysregulation is necessary for the preferential apoptotic response. Second, persistent recipient‐derived blood leukocytes are consistent with incomplete marrow ablation and partial donor reconstitution despite the reported conditioning regimen. Marrow ablation, bone‐marrow chimerism and recipient progenitors were not directly assessed, so the basis of incomplete donor replacement remains unresolved. The separate donor‐genotype cohorts do not provide a direct head‐to‐head comparison of Tet2^+/+^ and Tet2^−/−^ donor stem‐cell fitness. Accordingly, interpretation is limited to the observed peripheral‐blood donor chimerism and donor‐compartment lineage frequencies. Cardiac CD68 staining in this model quantifies total macrophage accumulation but cannot distinguish donor‐derived *Tet2*‐deficient cells from recipient‐derived or tissue‐resident macrophages; lineage‐resolved cardiac immune profiling will therefore be necessary to define the effects on mutant‐cell tissue infiltration. Finally, the narrow therapeutic index and dose‐dependent cardiotoxicity of cardiac glycosides limit the direct translational application of Lanatoside C. Recent experimental evidence further demonstrates concentration‐dependent cardiomyocyte injury associated with mitochondrial depolarization and apoptotic signaling following Lanatoside C exposure, as well as impaired cardiac function following prolonged high‐dose administration *in* vivo [54]. Importantly, differential Lanatoside C sensitivity was reproduced both following experimental TET2 reduction in primary human macrophages and independently in sequencing‐confirmed *TET2*‐mutant patient‐derived macrophages. Although the estimated IC_50_ in TET2‐mutant patient‐derived macrophages was numerically comparable to early post‐dose serum concentrations reported in a historical human intravenous study of Lanatoside C, these historical measurements were obtained shortly after dosing and do not define a modern therapeutic exposure range; the present data therefore do not establish a clinically usable therapeutic window [45, 46, 47]. Accordingly, Lanatoside C should be regarded in the present study as a clinically annotated mechanistic probe of a TET2‐associated ion‐mitochondrial vulnerability, rather than as a candidate for immediate clinical repurposing. Translation of the identified vulnerability will require rigorous pharmacokinetic, electrophysiological and long‐term safety assessment, together with the development of safer analogues, intermittent‐exposure regimens or myeloid‐targeted delivery strategies. In parallel, emerging approaches for phenotypic risk stratification may help identify CHIP carriers with greater residual cardiovascular risk and thereby support future genotype‐informed prevention and intervention strategies [55]. Complete blood counts, hemoglobin concentrations, erythroid indices and absolute blood‐cell counts were not systematically assessed in the transplantation cohort. Survival and the absence of overt adverse events cannot exclude subclinical anemia or other cytopenias, and the present study does not establish hematological safety.

More broadly, these findings suggest that clonal hematopoiesis mutations may create genotype‐specific metabolic liabilities that can be pharmacologically exploited. Whether analogous vulnerabilities exist in other CH drivers, such as DNMT3A or ASXL1, and whether targeting downstream myeloid effector‐cell states can alter long‐term clonal behavior, remain important questions for future study.

In summary, this study identifies a calcium‐associated ion‐mitochondrial vulnerability in TET2 loss‐of‐function myeloid cells and shows that pharmacological amplification of this state can preferentially suppress pathogenic macrophage activity. By integrating chemical‐genetic profiling, human multicellular cardiac modelling and in vivo validation, these findings support genotype‐informed targeting of myeloid effector programmes as a potential strategy to attenuate CH‐associated cardiac inflammation.

## 4 Experimental Section

### 4.1 Human Myeloid Cell Culture

Human myeloid cells (THP‐1) were obtained directly from Deutsche Sammlung von Mikroorganismen und Zellkulturen (ACC16, DSMZ; established from the peripheral blood of a 1‐year‐old boy). No additional in‐house cell‐line authentication was performed. Cells between passages 3 and 7 were used for all experiments. Cells were cultured in RPMI‐1640 medium (Gibco, A1049101) supplemented with 10% FCS (Gibco, 10270‐106), 1% Penicillin‐Streptomycin (Sigma–Aldrich, P4333‐100 mL), and L‐Glutamine (Sigma–Aldrich, G7513). THP‐1 cells were maintained at 0.2‐1.0 × 10^6^ cells mL^−1^ and split 1:2 to 1:3 every 3–4 days.

### 4.2 CDM Dissociation Assay

A total of 40 CDMs were collected in a 1.5 mL tube, and 1 mL of 2.5% trypsin (Merck, 59427C‐100ML) was added. The tube was then centrifuged at 1400 rpm for 15 min at 37°C (Eppendorf Thermomixer Compact Next). The cell pellet was pipetted up and down several times until most cells were dissociated and then centrifuged for another 5 min. One milliliter of culture medium was added to stop the dissociation reaction.

### 4.3 Macrophage Differentiation

THP‐1 monocytes were seeded in a 6‐well plate (Thermo Fisher Scientific, 140675) at a density of 5 × 10^5^ cells per well. Phorbol 12‐myristate 13‐acetate (PMA; Sigma‐Aldrich, P1585) was added to each well to reach a final concentration of 100 ng mL^−1^. The medium was replaced with fresh medium without PMA after 48 h at 37°C in 5% CO_2_. Cells were incubated for a further 24 h at 37°C in 5% CO_2_ to allow differentiation into macrophages. Trypsin (Gibco, 2215674) was used for macrophage detachment prior to cell splitting.

### 4.4 *TET2*‐KD Macrophage‐CDM Co‐Culture Model

Human TET2 siRNA (siTET2) and non‐targeting control pool siRNA (siScr) were purchased from Horizon (L‐013776‐03‐0020 and D‐001810‐10‐20, respectively). For transfection, 500 µL Opti‐MEM (Gibco, 31985062), 2 µL siRNA (100 pmol), and 6.25 µL LTX PLUS reagent (Invitrogen, 15338100) were mixed and incubated at room temperature for 15 min. Lipofectamine LTX (6.25 µL; Invitrogen, 15338100) was then added and the mixture was incubated for a further 30 min before addition to each well containing 1 × 10^5^ THP‐1‐derived macrophages in 2 mL culture medium. Cells were incubated for 18 h at 37°C and 5% CO_2_ before downstream experiments.

To establish the macrophage‐CDM co‐culture model, 5000 siScr or siTET2 macrophages were added to individual CDMs and co‐cultured for 48 h. CDMs were then collected and extensively washed to remove non‐adherent cells before downstream analysis. All siRNA sequences are listed in Extended Data Table S2.

### 4.5 RNA Isolation, Reverse Transcription and qRT‐PCR

For each biological replicate, 100 CDMs or 1 × 10^^6^ siScr or siTET2 macrophages treated with vehicle or Lanatoside C were harvested in TRIzol (Invitrogen). Total RNA was isolated using the RNeasy Mini Kit (Qiagen, 74106) according to the manufacturer's instructions. RNA was reverse transcribed into cDNA with the RNA to cDNA EcoDry Premix (random hexamers) kit (Clontech, 639545). Quantitative real‐time PCR (qRT‐PCR) was performed using iTaq Universal SYBR Green Supermix (Bio‐Rad, 1725121) on a PikoReal Real‐Time PCR system (Thermo Fisher Scientific). Cycle threshold (CT) values were normalized to the housekeeping gene HPRT. Primer sequences are provided in Extended Data Table S3.

### 4.6 Chemical‐Genetic Synthetic Lethality Screen

Macrophages were seeded in 384‐well plates at a density of 2,500 cells per well (Greiner Bio, 781186). Cells were subsequently treated with compounds from the Anti‐Cardiovascular Disease Compound Library (TargetMol, L5400; Extended Data Table S1), which were added at 10 µM; 1% DMSO (Carl Roth, A994.2) was used as a vehicle control. After 48 h, cell viability was measured using the Alamar Blue assay (Bio‐Rad, BUF012B).

For hit selection, viability values were normalized to the genotype‐matched DMSO vehicle control. Compounds were considered genotype‐selective hits if they preserved siScr macrophage viability above 80% of the siScr vehicle control while reducing siTET2 macrophage viability below 70% of the siTET2 vehicle control. Compounds fulfilling these criteria were ranked by the difference in viability between siScr and siTET2 macrophages and selected for secondary validation.

### 4.7 Animal Experiments

All animal experiments were conducted at the Barrier Facility of the Laboratory Animal Center of Jilin University (SYXK(Ji)2016‐0001) under the supervision of the Institutional Animal Care and Use Committee (IACUC No. SY202402001) and in compliance with institutional and national ethical guidelines for animal welfare. Animals were housed under specific pathogen‐free (SPF) conditions according to GB14925 standards, with free access to food and water.

Bone marrow transplantation (BMT) was performed in separate Tet2 donor‐genotype cohorts; the measured outcome was partial donor‐cell reconstitution. Bone marrow hematopoietic cells were isolated from male *Tet2*‐deficient donor mice (C57BL/6Smoc‐Tet2em1Smoc, CD45.2^+^) or male Tet2 wild‐type donor mice (C57BL/6, CD45.2^+^). Male CD45.1^+^ recipient mice (C57BL/6Smoc‐Ptprcem1(K302E)Smoc, 6–8 weeks old) underwent total‐body irradiation using an X‐RAD 320 irradiator. A total irradiation dose of 11 Gy was delivered in two fractions of 5.5 Gy separated by 4 h. Following completion of the second irradiation fraction, the first donor‐cell transplantation was initiated on the same day. Tet2^−/−^ and Tet2^+/+^ donor bone marrow cells were transplanted separately and were not mixed before transplantation. Each recipient mouse received donor cells from only one genotype. Donor bone marrow cells were prepared as single‐cell suspensions and administered intravenously at 5 × 10^6^ cells per day for three consecutive days, corresponding to a cumulative dose of 1.5 × 10^7^ donor cells per recipient. Following irradiation and transplantation, mice were maintained under standard SPF husbandry conditions with free access to food and water and underwent enhanced monitoring for general condition, body weight, food and water intake, survival, and overt adverse events. No recipient mice were excluded from the peripheral‐blood analyses, and no attrition occurred during the transplantation or treatment periods.

Four weeks after transplantation, before the initiation of Lanatoside C treatment, peripheral blood was collected to assess baseline hematopoietic reconstitution and donor‐cell chimerism by flow cytometry. Donor‐derived leukocytes were defined as CD45.2^+^ cells among total live circulating CD45^+^ leukocytes, whereas residual recipient‐derived cells were identified as CD45.1^+^ cells. The donor inocula contained only CD45.2^+^ donor marrow, so CD45.1^+^ leukocytes were recipient‐derived. Donor chimerism was calculated as the percentage of CD45.2^+^ donor‐derived cells among total live CD45^+^ leukocytes. Peripheral blood was collected again after the one‐month treatment period, and total donor chimerism was reassessed using the same CD45.1/CD45.2 gating strategy and denominator. Pre‐ and post‐treatment measurements were obtained from the same recipient mice, allowing paired assessment of changes in total circulating CD45.2^+^ donor‐derived leukocyte representation over the treatment interval. These measurements assess peripheral‐blood leukocyte chimerism; marrow ablation, bone‐marrow chimerism and recipient progenitors were not directly assessed. Persistent recipient‐derived blood leukocytes were interpreted as consistent with incomplete marrow ablation and partial donor reconstitution, not as direct measurement of the extent or mechanism of marrow recovery. For each mouse and lineage in Figure 5D–K, the underlying lineage frequency was defined within the circulating live CD45.2^+^ donor‐derived leukocyte compartment. The displayed relative frequency was calculated as 100 × (individual‐mouse lineage frequency / mean lineage frequency in the reference group). The reference was the Tet2^+/+^ donor‐cell cohort at baseline (Figure 5D; *n* = 16 mice per donor‐genotype cohort before treatment allocation) or the Tet2^+/+^ vehicle group at the endpoint (Figure 5E–K; *n* = 8 mice per treatment group). Thus, the reference‐group mean was set to 100%, but the individual control values retained their between‐mouse variability. These normalized values are distinct from the unnormalized flow‐cytometric lineage percentages and absolute blood‐cell counts.

After baseline chimerism assessment at four weeks post‐transplantation, mice were manually randomized within each donor‐genotype cohort to vehicle or Lanatoside C treatment (*n* = 8 per group), resulting in four groups: Tet2^+/+^ vehicle, Tet2^+/+^ Lanatoside C, Tet2^−/−^ vehicle, and Tet2^−/−^ Lanatoside C. Treatment administration was performed by an investigator blinded to group allocation. Lanatoside C was freshly prepared in 0.9% NaCl immediately before administration and administered intraperitoneally at 5 mg/kg/day in a final injection volume of 200 µL per mouse for one month (approximately weeks 4–8 after transplantation). Endpoint sampling was performed at approximately week 8. Vehicle‐control mice received an equivalent volume (200 µL) of 0.9% NaCl on the same schedule. Tolerability was monitored throughout the treatment period by assessment of body weight, general condition, activity, food and water intake, survival, and overt adverse events.

No overt treatment‐related adverse events or mortality were observed. All 32 recipient mice completed the study, with no exclusions or attrition. Complete blood counts, hemoglobin concentrations, erythroid indices and absolute blood‐cell counts were not systematically measured; subclinical anemia or other cytopenias cannot therefore be excluded.

### 4.8 Cardiac Whole‐Mount Immunofluorescence and Quantification

Cardiac whole‐mount immunofluorescence was performed on ventricular tissues available from a subset of the murine bone marrow transplantation cohort. Although peripheral blood flow‐cytometry analyses were performed using *n* = 8 mice per group, ventricular tissues were available for whole‐mount analysis from *n* = 4 mice per group, as the remaining cardiac tissues had been allocated to pre‐specified collaborative downstream analyses. Hearts were fixed in 4% paraformaldehyde, and ventricular myocardial preparations were processed for whole‐mount immunofluorescence without cryosectioning. Samples were permeabilized with 0.1% Triton X‐100 in PBS, blocked with 5% serum in PBS, and incubated overnight at 4°C with anti‐CD68 antibody (Abcam, ab53444, 1:50, Extended Data Table S4) and anti‐α‐actinin antibody (Sigma–Aldrich, A7811, 1 µg mL^−1^, Extended Data Table S4). After washing, samples were incubated with the appropriate fluorescence‐conjugated secondary antibodies for 1 h at room temperature, counterstained with DAPI, and mounted in antifade mounting medium. Confocal images were acquired using identical microscope settings across all groups. Representative single optical sections are shown in Figure 5M. For quantification, three non‐overlapping fields of ventricular myocardium were analyzed per mouse. CD68^+^ cells were counted using ImageJ, and the mean CD68^+^ cell number from each mouse was treated as one biological replicate. Values were normalized to the Tet2^+/+^ vehicle group, which was set to 100%.

### 4.9 Isolation of Primary human CD14^+^ Monocytes

Peripheral blood samples used for the primary‐human‐macrophage experiments were obtained through the Technical University of Munich. All samples were collected and processed in accordance with the relevant institutional ethical requirements and informed‐consent procedures. The institutional review board and Ethics Committee of the Technical University of Munich, Germany, approved the work on CHIP (273/18 S—27 June 2018; 2018‐325‐S‐KK—22 August 2018; 2023‐54‐S‐KK—24 March 2023). All studies were conducted in accordance with the provisions of the Declaration of Helsinki and the International Conference on Harmonization guidelines for good clinical practice.

For the experiments shown in Extended Data Figure S12A–C, peripheral blood mononuclear cells (PBMCs) were isolated from buffy coats obtained from clinically healthy donors. PBMCs (1 × 10^7^ cells per isolation) were washed and resuspended in ice‐cold PBS supplemented with 2% FBS. CD14^+^ monocytes were purified using Dynabeads FlowComp Human CD14 (Thermo Fisher Scientific, 11367D) according to the manufacturer's protocol. Briefly, PBMCs were incubated with FlowComp Human CD14 antibody (25 µL per 5 × 10^7^ PBMCs) for 10 min at 4°C with gentle inversion, followed by washing (350 × g, 8 min, 4°C) and resuspension in cold isolation buffer. Washed FlowComp Dynabeads (75 µL per 5 × 10^7^ PBMCs) were added, and the suspension was gently mixed and incubated for 15 min at 4°C. Bead‐bound CD14^+^ cells were magnetically separated, washed twice in cold buffer, and released using FlowComp Release Buffer (1 mL per isolation) according to the manufacturer's instructions. After magnetic removal of residual beads, the bead‐free monocytes were centrifuged (350 × g, 8 min, 4°C) and resuspended in complete culture medium for downstream macrophage differentiation.

### 4.10 Differentiation of Primary Human Monocytes Into Macrophages

Bead‐free CD14^+^ monocytes were cultured in RPMI‐1640 supplemented with 10% FBS and 1% Penicillin‐Streptomycin and differentiated into macrophages using recombinant human M‐CSF (STEMCELL Technologies, 78150). Lyophilized M‐CSF was reconstituted to ≥ 0.1 mg/mL in sterile water according to the manufacturer's instructions and stored at ‐20°C to ‐80°C until use. Monocytes were seeded at 0.5‐1 × 10^6^ cells mL^−1^ and differentiated for 7 days in the presence of 50 ng mL^−1^ M‐CSF, with medium replenished every 2–3 days. On day 7, adherent macrophages were maintained in culture with 20 ng mL^−1^ M‐CSF.

For the siRNA‐based primary‐human‐macrophage experiments shown in Extended Data Figure S12A–C, macrophages derived from four independent healthy human donors were transfected with siScr or siTET2 using the Lipofectamine LTX‐based transfection procedure described above. siScr and siTET2 conditions were paired within each donor, with each donor treated as one biological replicate. Following transfection, macrophages were exposed to the indicated concentrations of Lanatoside C for 48 h before assessment of cell viability and apoptosis.

### 4.11 Patient‐Derived Macrophage Studies

Independent patient‐derived peripheral blood samples were used for the experiments shown in Extended Data Figure S12D,E. TET2 mutation status had been determined by sequencing by the providing center before sample provision. Patients were stratified into TET2‐mutant and TET2‐WT comparator groups according to this pre‐existing sequencing information. The TET2‐WT comparator group was selected to match the TET2‐mutant group for age and sex. Eight independent individuals were included in each group, with each individual treated as one biological replicate. All patient‐derived samples were collected and processed in accordance with the relevant institutional ethical requirements and informed‐consent procedures. The institutional review board and Ethics Committee of the Technical University of Munich, Germany, approved our work on CHIP (273/18 S—27 June 2018; 2018‐325‐S‐KK—22 August 2018; 2023‐54‐S‐KK—24 March 2023). All studies were conducted in accordance with the provisions of the Declaration of Helsinki and the International Conference on Harmonization guidelines for good clinical practice.

PBMCs were isolated from peripheral blood, and CD14^+^ monocytes were purified and differentiated into macrophages using the same CD14^+^ isolation and M‐CSF‐based differentiation procedures described above. Importantly, patient‐derived macrophages were not subjected to experimental siRNA‐mediated TET2 knockdown. Following differentiation, macrophages were treated with Lanatoside C at 0, 0.1, 0.2, 0.3, 0.5, 1.0, 2.0, 5.0, 7.5 or 10 µM for 48 h before viability assessment and concentration‐response modelling.

### 4.12 Qtracker Labelling

Macrophages were differentiated from THP‐1 monocytes, then treated with siRNAs (18 h incubation), washed, and labelled with Qtracker 655 Cell Labeling Kit (Thermo Fisher Scientific, Q25021MP) for 1 h according to the manufacturer's instructions, washed with PBS, and dissociated with 0.05% trypsin. Macrophages were then added to CDMs (5,000 macrophages per 1 CDM) and cultured in 37°C / 5% CO_2_. Cultures were maintained in 96‐well U‐bottom plates.

### 4.13 Trypan Blue Assay

Cell viability was measured by mixing 0.4% Trypan blue solution (Gibco, 15250061) with an equal volume of myeloid cell suspension, followed by counting live (transparent) and dead (blue) cells under a microscope.

### 4.14 Calcium Transient Assay

For drug‐treatment experiments, CDMs were exposed to vehicle or 10 µM Lanatoside C for 48 h before calcium‐transient measurements. Following treatment, CDMs were incubated with labelling medium containing 2.5 µM Cal‐520 (Abcam, ab171868) and 0.04% Pluronic F‐127 (Thermo Fisher Scientific, P3000MP) for 90 min at 37°C and 5% CO_2_. CDMs were then washed twice with PBS and once with pre‐warmed culture medium and transferred individually to ultra‐low‐attachment black‐walled 384‐well plates (Corning, 4516), with one CDM per well in 100 µL culture medium. Calcium‐dependent fluorescence was recorded using a CLARIOstar plate reader (BMG Labtech) for 750 consecutive measurements at 0.02‐s intervals. Calcium‐transient amplitude and beating frequency were quantified from the recorded traces.

### 4.15 cTnT assay

Culture media from CDMs were collected and diluted 1:3 with NS Reagent from the Human Cardiac Troponin T ELISA Kit (Abcam, ab223860). Cardiac troponin T concentrations were measured according to the manufacturer's instructions. Absorbance was measured at 450 nm using a CLARIOstar plate reader (BMG Labtech).

### 4.16 Cytokine ELISA

IL‐1β, IL‐6, and total IL‐18 concentrations in macrophage monoculture supernatants and CDM co‐culture media were measured using human DuoSet ELISA kits from R&D Systems (Bio‐Techne) according to the manufacturer's instructions. Human IL‐1β was measured using the Human IL‐1β/IL‐1F2 DuoSet ELISA (DY201‐05; standard‐curve range, 3.91–250 pg/mL^−1^), IL‐6 using the Human IL‐6 DuoSet ELISA (DY206‐05; 9.38–600 pg mL^−1^), and total IL‐18 using the Human Total IL‐18 DuoSet ELISA (DY318‐05; 11.7–750 pg mL^−1^). Samples were diluted 1:3 before analysis, and concentrations interpolated from the standard curves were corrected for the corresponding threefold dilution factor. Cytokine concentrations were normalized to the siScr vehicle group unless otherwise stated.

### 4.17 Proliferation Assay

Macrophages were seeded in 96‐well plates at 2,500 cells per well. Cell proliferation was quantified using the CellTiter 96 AQueous One Solution Cell Proliferation Assay (Promega, G3852) according to the manufacturer's instructions. Absorbance was measured at 490 nm using a BMG Labtech plate reader.

### 4.18 Immunofluorescence Staining

CDMs were collected and fixed with 4% PFA (Santa Cruz, CAS 30525‐89‐4) overnight at 4°C. For whole‐mount staining, samples were permeabilized with 1% Triton X‐100 in PBS for 1 h, followed by blocking with 5% horse serum (Sigma–Aldrich, H0146) in PBS for 1 h at room temperature. Primary antibodies (Extended Data Table S4) were diluted in 2% horse serum and incubated overnight at 4°C. Samples were washed five times with PBS containing 1% Tween 20 and 0.002% Triton X‐100 for 10 min each at room temperature. Secondary antibodies (Extended Data Table S4) were diluted in 2% horse serum and incubated for 4 h at room temperature in the dark. After three additional washes, CDMs were mounted using ProLong Gold Antifade Mountant (Thermo Fisher Scientific, P36934) and imaged with a Leica TCS SP8 confocal microscope.

For dissociated and myeloid cells, fixation was performed using 4% cold PFA at 4°C for 10 min, followed by permeabilization with either 0.5% Triton X‐100 for cell‐surface staining or 1% Triton X‐100 for intracellular staining in PBS for 10 min at 4°C. Cells were then blocked in 5% horse serum in PBS for 1 h at room temperature. Primary antibodies were diluted in 2% horse serum and incubated overnight at 4°C. Cells were washed three times with PBS before incubation with fluorescence‐conjugated secondary antibodies diluted in 2% horse serum for 1 h at room temperature in the dark. Cells were then washed three times with PBS, mounted with ProLong Gold Antifade Mountant, and imaged using a Leica TCS SP8 confocal microscope. For TET2‐matched apoptosis staining, siScr and siTET2 THP‐1‐derived macrophages treated with vehicle or Lanatoside C were co‐stained with anti‐TET2 antibody (Invitrogen, MA5‐31442, 1:50) and anti‐cleaved Caspase‐3 antibody, together with DAPI nuclear counterstaining. Images were acquired using identical microscope settings across all groups.

Primary antibodies were prepared as follows: anti‐α‐Actinin (1 µg mL^−1^), anti‐cleaved Caspase‐3 (3 µg mL^−1^), anti‐PCM1 (1 µg mL^−1^), anti‐CD68 (3 µg mL^−1^), pan‐neuronal marker (4 µg mL^−1^), VE‐Cadherin (2 µg mL^−1^), anti‐COL1A1 (2 µg mL^−1^), anti‐ATP5A (1 µg mL^−1^), anti‐cytochrome c (3 µg mL^−1^), and anti‐TET2 (Invitrogen, MA5‐31442, 1:50), and samples were incubated overnight at 4°C. After washing, samples were incubated with the appropriate species‐specific Alexa Fluor 488‐, 555‐, 594‐, or 647‐conjugated secondary antibodies at a dilution of 1:500 for 1 h at room temperature, protected from light. DAPI (1:1000; Abcam, ab228549) was applied for nuclear staining and incubated for 1 h for cell staining or 4 h for CDM staining.

### 4.19 Fluorescence Quantification

Apoptosis and infiltration quantification was performed using ImageJ to analyze cleaved‐Caspase‐3‐positive and Qtracker‐positive areas, respectively. Measurements were acquired from maximum Z‐projections of non‐overlapping z‐stacks per organoid sample. For Figure 1J, cleaved‐Caspase‐3‐positive area was quantified specifically within α‐actinin‐positive cardiomyocyte regions. Three non‐overlapping cardiomorph areas were quantified for each biological replicate and averaged to generate a single replicate‐level value before inferential statistical testing. Six biologically independent replicates were analyzed per condition. An equal intensity threshold was applied to all images to ensure consistent binary conversion across conditions. Positive‐marker areas were segmented from the binary images and expressed as a fraction of the corresponding analyzed area. For Figure 1M, fibrosis was quantified from maximum Z‐projections by applying a consistent intensity threshold to the COL1A1 channel after background subtraction. COL1A1‐positive area was segmented and expressed as a fraction of the analyzed CDM area. For Extended Data Figure S9B, COL1A1 signal was quantified as mean fluorescence intensity after background subtraction. Identical acquisition and analysis settings were applied across all conditions. Cytochrome c redistribution was quantified using ImageJ by measuring cytochrome c fluorescence outside ATP5A‐positive mitochondrial regions. Identical intensity thresholds and analysis settings were applied across all conditions, and increased non‐mitochondrial cytochrome c signal was interpreted as being consistent with cytochrome c redistribution during apoptotic signaling.

### 4.20 Intracellular Calcium Fluorescence Assay

Intracellular Ca^2+^ levels in siScr and siTET2 macrophages treated with vehicle or 10 µM Lanatoside C for 48 h were assessed using the fluorescent calcium indicator Cal‐520 (Abcam, ab171868). Cells were incubated in labeling medium containing 2.5 µM Cal‐520 and 0.04% Pluronic F‐127 (Thermo Fisher Scientific, P3000MP) in PBS for 90 min at 37°C and 5% CO_2_. Following dye loading, cells were washed twice with PBS and once with pre‐warmed culture medium. Cal‐520 fluorescence was subsequently measured as a single endpoint readout using a CLARIOstar plate reader (BMG Labtech). Fluorescence values were normalized to the siScr vehicle group and expressed as relative intracellular Ca^2+^ fluorescence.

### 4.21 Mitochondrial Rhod‐2 Calcium Imaging

THP‐1‐derived macrophages transfected with siScr or siTET2 were treated with vehicle or 10 µM Lanatoside C for 48 h. Following treatment, cells were loaded with 5 µM Rhod‐2 AM (Invitrogen, R1245MP) for 30 min at 37°C in the dark and washed twice with pre‐warmed culture medium. Cells were subsequently labelled with 100 nM MitoTracker Green FM (Invitrogen, M7514) before live‐cell imaging, and Hoechst 33342 (1 µg mL^−1^) was added during the final 15 min of the labelling period. Live‐cell images were acquired using a Leica TCS SP8 confocal microscope with identical acquisition settings across all conditions.

Rhod‐2 fluorescence was quantified using Fiji/ImageJ. Cellular Rhod‐2 fluorescence was quantified on a per‐cell basis, whereas mitochondrial‐enriched Rhod‐2 fluorescence was determined as the mean Rhod‐2 intensity within MitoTracker Green‐positive regions. For both cellular and mitochondrial‐enriched Rhod‐2 analyses, measurements from individual cells were averaged within each biological replicate to generate a single replicate‐level value before inferential statistical testing.

### 4.22 JC‐10 and Cleaved‐Caspase‐3 Co‐Staining

JC‐10 (ENZO, ENZ‐52305) was used to assess mitochondrial membrane potential according to the manufacturer's instructions. Briefly, JC‐10 was added to vehicle‐ or Lanatoside C‐treated siScr and siTET2 THP‐1‐derived macrophages at 15 µM for 1 h at 37°C. After incubation, cells were washed once with PBS before fluorescence imaging. Cleaved Caspase‐3 antibody was obtained from Abcam (Extended Data Table S4) and used at 1:100; Alexa Fluor 647 secondary antibody (Invitrogen, A‐21245) and DAPI were used at 1:1,000.

### 4.23 Western Blotting

Macrophages were harvested and lysed in RIPA buffer (Thermo Fisher Scientific, 89901) containing 25 mM Tris‐HCl (pH 7.6), 150 mM NaCl, 1% NP‐40, 1% sodium deoxycholate, and 0.1% SDS, supplemented with a protease and phosphatase inhibitor cocktail (Thermo Fisher Scientific, 78444). Protein concentrations were determined using the BCA Protein Assay Kit (Thermo Fisher Scientific, 23225). Equal amounts of protein were resolved on SDS‐PAGE gels (Bio‐Rad, 4561083) and subsequently transferred onto 0.45 µm PVDF membranes (Millipore, IPVH00010). PageRuler Prestained Protein Ladder (Thermo Fisher Scientific, 26616) and Precision Plus Protein All Blue Prestained Protein Standards (Bio‐Rad, 1610373) were used as molecular‐weight standards.

Membranes were blocked with 5% skim milk in PBS containing 0.05% Tween 20 (PBS‐T) for 1 h at room temperature. Following blocking, membranes were incubated overnight at 4°C with primary antibodies against GAPDH (0.5 µg/mL), cleaved Caspase‐3 (3 µg mL^−1^), ATP5A (1 µg mL^−1^), Cytochrome c (2 µg mL^−1^), TET2 C‐terminus (Cell Signaling Technology, #45010, 1:500), or TET2 N‐terminus (Abcam, ab230358, 1:500) on a shaking platform at 70 rpm. For validation of TET2 knockdown, TET2 protein expression was assessed using antibodies recognizing both the C‐terminal and N‐terminal regions of TET2. The C‐terminal TET2, N‐terminal TET2, and GAPDH signals were detected on the same PVDF membrane, with stripping and re‐probing performed when necessary.

The membranes were then washed with PBS‐T and incubated with horseradish peroxidase‐conjugated secondary antibodies (Goat Anti‐Rabbit IgG H&L, Abcam, ab6721; Goat Anti‐Mouse IgG H&L, Abcam, ab205719) at 1:10,000 for GAPDH and 1:5,000 for ATP5A, Cytochrome c, cleaved Caspase‐3, and TET2, for 1 h at room temperature. Immunoreactive bands were detected using SuperSignal West Femto Substrate (Thermo Fisher Scientific, 34096). When sequential detection was required, membranes were stripped using Western Blot Stripping Buffer (Thermo Fisher Scientific, 21059) and re‐probed. Band intensities were quantified using ImageJ software (National Institutes of Health, USA). TET2 protein levels detected with the C‐terminal and N‐terminal antibodies were normalized to GAPDH and expressed relative to siScr controls.

### 4.24 EdU Staining

EdU incorporation was assessed using the Click‐iT EdU imaging kit (Invitrogen, C10337) according to the manufacturer's instructions. THP‐1‐derived macrophages were exposed to vehicle (DMSO) or Lanatoside C in the presence of 10 µM EdU for 48 h at 37°C before fixation, detection, and imaging.

### 4.25 RNA Sequencing and Bioinformatic Analysis

Total RNA was subjected to quality control before library preparation. RNA concentration and purity were assessed by NanoDrop spectrophotometry, RNA integrity was evaluated using an Agilent 2100 Bioanalyzer or equivalent automated electrophoresis platform, and sample quality was additionally assessed by agarose gel electrophoresis. Poly(A)‐enriched mRNA libraries were prepared by Novogene (UK) and sequenced on an Illumina NovaSeq 6000 platform using 150‐bp paired‐end sequencing (PE150), with a target output of approximately 12 Gb raw sequence data per sample and a sequencing‐quality specification of Q30 ≥ 85%. FASTQ files were subsequently processed and analyzed using Partek Flow v10 (Partek Inc., St. Louis, MO, USA). Reads were aligned to the human GRCh38/hg38 reference genome using STAR v2.7, and gene‐level counts were generated using featureCounts. Lowly expressed genes, defined as genes with counts ≤ 1 across all samples, were excluded from downstream analysis. Expression values were normalized to counts per million (CPM) and transformed as log_2_ (CPM + 4). Differential‐expression analysis was performed using Gene Specific Analysis (GSA), with experimental group specified as a categorical factor and pairwise contrasts performed relative to the siScr vehicle reference group where indicated. Nominal *p* values derived from GSA were used for exploratory transcriptomic comparisons; no multiple‐testing correction was applied. Three biologically independent replicates per condition were included in the downstream analyses. For heatmap visualization, expression values were standardized across genes by Z‐score transformation. Three‐dimensional UMAP was performed on the filtered log_2_ (CPM + 4)‐transformed expression matrix using all genes retained after low‐expression filtering, with three output dimensions, five nearest neighbors, a minimum distance of 0.3, cosine distance, and a fixed random seed of 42.

### 4.26 IC_50_ Estimation

IC_50_ values were estimated using a four‐parameter logistic regression model [56]. Lanatoside C concentration‐response analyses were performed in two complementary primary human macrophage systems.

For the siRNA‐based primary‐human experiments shown in Extended Data Figure S12A–C, monocyte‐derived macrophages from four independent human donors were transfected with siScr or siTET2 and treated with 0, 1, 2, 4, 6, 8, 10 or 15 µM Lanatoside C for 48 h. Cell viability was assessed using the Alamar Blue assay, and apoptosis was assessed using the Caspase‐3 activity assay (Biotium, 30009‐2). Each donor was treated as one biological replicate.

For the independent patient‐derived experiments shown in Extended Data Figure S12D,E, macrophages from eight individuals per group were treated with 0, 0.1, 0.2, 0.3, 0.5, 1.0, 2.0, 5.0, 7.5 or 10 µM Lanatoside C for 48 h. Cell viability was assessed using the Alamar Blue assay. Patient‐derived macrophages were analyzed according to sequencing‐confirmed TET2 mutation status and were not subjected to experimental siRNA‐mediated TET2 knockdown.

For the siRNA‐based primary‐human experiments, concentration‐response values were normalized to the corresponding siScr vehicle control, which was set to 100%. For the patient‐derived experiments, concentration‐response values were expressed relative to the mean vehicle response of the TET2‐WT comparator group, which was set to 100%.

All concentration‐response data were normalized to the siScr vehicle control, which was set to 100%. Concentration‐response curves were fitted using a four‐parameter logistic regression model, from which IC50 values were estimated by the following equation:

$$Y=Bottom+\frac{Top-Bottom}{1+{\left(\frac{X}{IC_{50}}\right)}^{Hill\;slop}}$$

where Bottom and Top are the fitted lower and upper asymptotes, respectively, X is the Lanatoside C concentration, IC_50_ is the concentration producing the half‐maximal fitted response, and the Hill slope describes the steepness of the concentration‐response relationship. Top, Bottom, IC_50_ and Hill slope were fitted without parameter constraints. 95% confidence intervals for IC_50_ estimates were determined using profile‐likelihood confidence intervals, and goodness‐of‐fit was assessed using R^2^. Donor‐level source values and complete curve‐fit parameters are provided in the Source Data. Data are presented as mean ± SEM.

### 4.27 Statistical Analysis

Statistical analyses were performed using GraphPad Prism 9. Unless otherwise specified, the biological replicate, rather than individual cells, wells, imaging fields, regions of interest, or organoids, was treated as the independent statistical unit. Technical replicates and nested measurements, including measurements from multiple cells, wells, imaging fields, regions, or organoids within the same biological replicate, were averaged to generate a single replicate‐level value before inferential statistical testing. The primary‐human concentration‐response experiments shown in Extended Data Figure S12A,B were analyzed separately using mixed‐effects models fitted by restricted maximum likelihood. Concentration was treated as a categorical fixed effect, together with siRNA condition and the concentration‐by‐siRNA‐condition interaction, and donor was included as a random effect to account for repeated measurements within the same individual. The patient‐derived viability data shown in Extended Data Figure S12D were analyzed using an analogous mixed‐effects model with concentration, TET2 mutation status and their interaction as fixed effects and participant as a random effect. Šídák‐adjusted planned comparisons were used to compare siScr with siTET2 macrophages, or *TET2*‐WT with *TET2*‐mutant macrophages, at each concentration. Four‐parameter logistic regression was used to estimate IC_50_ values and was not used as the primary inferential test of differences between groups. For Figure 5C, paired measurements at week 4 and approximately week 8 were analyzed using two‐way repeated‐measures ANOVA followed by Šídák's multiple‐comparisons test, as specified in the figure legend. Comparisons between two independent groups, including the baseline donor‐genotype comparison in Figure 5D, were performed using two‐tailed unpaired t‐tests with Welch's correction. Except for the repeated‐measures donor‐chimerism and donor‐ and participant‐level concentration‐response analyses described above, experiments involving two independent variables were analyzed using two‐way ANOVA followed by Tukey's multiple‐comparisons test. Exact statistical tests and post hoc comparisons are specified for the corresponding figure panels. Data are presented as mean ± SEM unless otherwise stated, and *p* < 0.05 was considered statistically significant. No statistical method was used to predetermine sample size.

## Author Contributions

**Y.W**. and **J.K**. conceived the study and designed the experimental strategy. **Y.W**. performed the experiments, analyzed and interpreted the data, prepared all figures, and wrote the manuscript. **Y.H**. performed and coordinated the animal experiments. **T.Y**., **M.D.P**., **A.P.D**., **M.W**., **C.Z**., **D.S**., **T.T.D**., **J.Kaur**, and **M.D**. contributed scientific discussion and technical input. **J.Krefting**, **M.Schwab**, **W.A**., and **M.S**. contributed to the acquisition and coordination of human samples, methodological guidance for CD14^+^ monocyte isolation and the primary‐human studies, interpretation of the human data, and critical revision of the manuscript. **J.W**., **A.M.Z**., and **S.D**. provided scientific guidance and contributed to data interpretation. **J.K**. supervised the study, contributed to data interpretation and critically revised the manuscript. All authors reviewed and approved the final manuscript.

## Conflicts of Interest

M.D.P. and J.K. are named inventors on a pending patent application relating to cardiac organoids. The remaining authors declare no competing interests.

## Supporting information




**Supporting File**: advs78748‐sup‐0001‐SuppMat.pdf.

## Data Availability

The raw and processed bulk RNA‐sequencing data generated in this study have been deposited in the NCBI Gene Expression Omnibus under accession number GSE344192 and are publicly available. All other data supporting the findings of this study are available within the article and its Supporting Information. Analysis scripts used for RNA‐sequencing processing, statistical analysis, and data visualization are available from the corresponding author upon reasonable request.
