# Supplementary material for: Targeting a Calcium‐Associated Mitochondrial Vulnerability in TET2‐Deficient Myeloid Cells
Source: Adv Sci (Weinh). 2026 Nov 9:e78748. Online ahead of print. doi: 10.1002/advs.78748 (PMC13748904; doi:10.1002/advs.78748)
Supplement: Supplementary file 1 [file ADVS-9999-e78748-s001.pdf]

Supporting Information

Targeting a calcium-associated mitochondrial vulnerability in TET2-deficient myeloid cells

Yue Wang[1,2,5], Minh Duc Pham[1,2,3], Despina Stefanoska[3], Ting Yuan[1,2,5], Arka Provo Das[1,2,5], Chaonan Zhu[1,2,5], Meiqian Wu[1,2], Thanh Thuy Duong[3], Jaskiran Kaur[1], Yu Hao[4], Johannes Krefting[7,8], Marius Schwab[7,8], Jonathan Ward[3], Maria Duda[3], Wesley Abplanalp[2,5,6], Moritz von Scheidt[7,8], Andreas M. Zeiher[1,2,5,6], Stefanie Dimmeler[2,5,6], and Jaya Krishnan[1,2,3,5,6]*

Extended Data Figures 1–12
Extended Data Tables 1–4

Extended Data Fig. 1

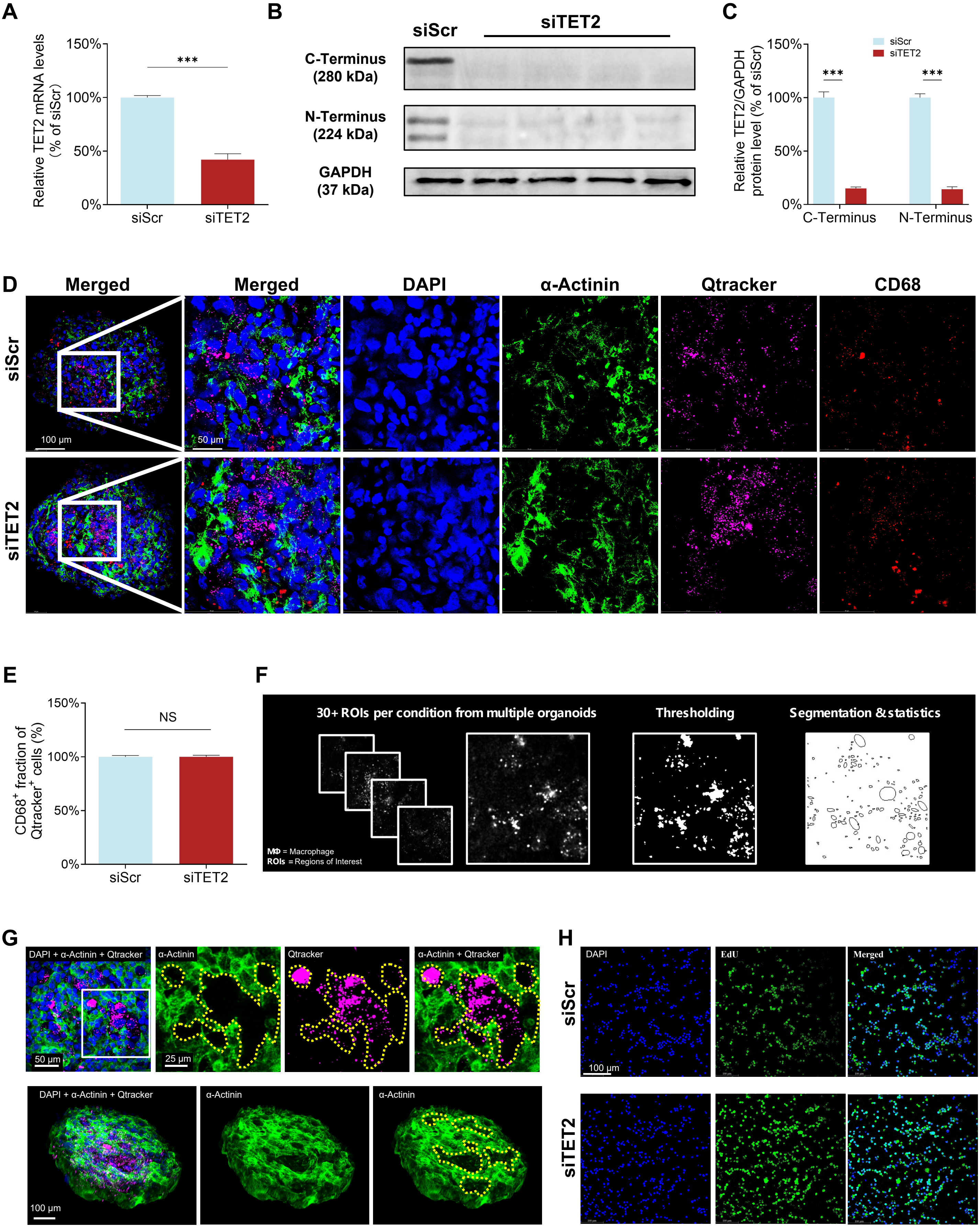

Extended Data Figure 1 | Characterization of the TET2-KD macrophage-cardiomorph co-culture model.

(A) Relative TET2 mRNA levels in CDMs infiltrated with siScr or siTET2 macrophages. n = 4 biologically independent replicates per condition.

(B) Representative Western blots showing TET2 protein expression detected with antibodies against the C-terminal and N-terminal regions of TET2. For the N-terminal blot, the upper band of the doublet was quantified. GAPDH served as the loading control.

(C) Densitometric quantification of TET2 protein levels normalized to GAPDH and expressed as percentage of siScr controls. TET2 protein levels were reduced to 15.0% and 14.3% of siScr controls using the C-terminal and N-terminal antibodies, respectively. n = 4 biologically independent replicates per condition.

(D) Representative immunofluorescence images showing co-localization of Qtracker-labelled infiltrating macrophages with CD68 within CDMs after 48 h of co-culture. Nuclei were stained with DAPI (blue), cardiomyocytes with α-Actinin (green), exogenously added macrophages with Qtracker (magenta), and macrophage marker expression with CD68 (red). Scale bar, 50-100 µm.

(E) Quantification of the proportion of Qtracker-positive cells that were also CD68-positive within CDMs. n = 6 biologically independent samples. Data are shown as mean ± SEM.

(F) Workflow for quantification of macrophage-infiltrated areas by thresholding, binary conversion, and automated segmentation.

(G) Representative immunofluorescence images of Qtracker-stained macrophages infiltrating CDMs. Nuclei were stained with DAPI (blue), cardiomyocytes with α-Actinin (green), and infiltrating macrophages with Qtracker (magenta). Scale bar, 25-50 µm.

(H) Representative immunofluorescence images of EdU-stained siScr and siTET2 macrophages. Nuclei were stained with DAPI (blue), and EdU was visualized in green. Scale bar, 100 µm.

Data are shown as mean ± SEM where applicable. Statistical significance in **(A,C)** was assessed using two-tailed unpaired t-tests with Welch's correction. $^{*}p < 0.05$, $^{**}p < 0.01$, $^{***}p < 0.001$, $^{****}p < 0.0001$.

Extended Data Fig. 2

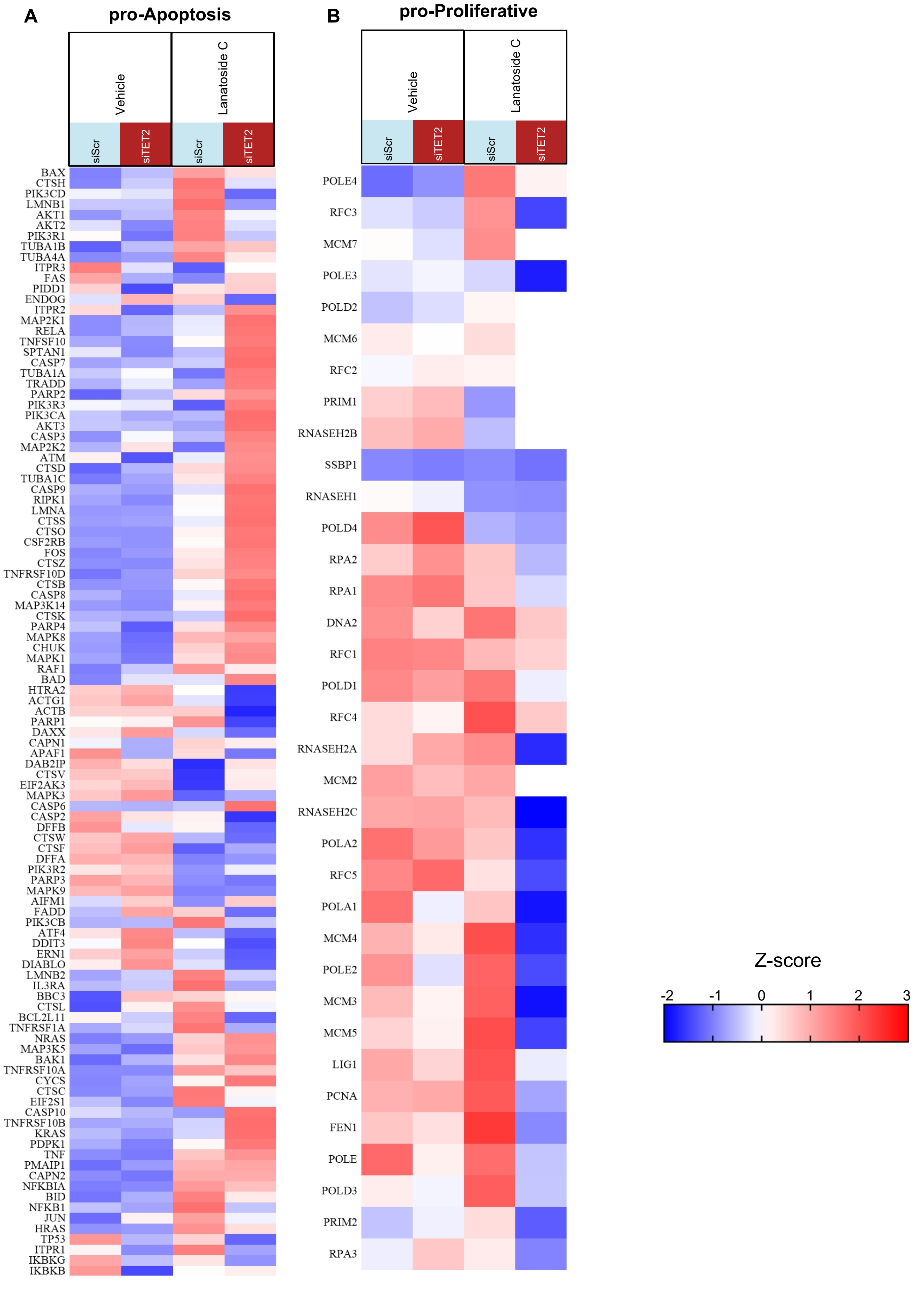

Extended Data Figure 2 | Extended expression analysis of bulk RNA-sequencing data from control and *TET2*-knockdown THP-1-derived macrophages.

(A) Heatmap of pro-apoptotic marker expression in siScr or siTET2 macrophages treated with vehicle or Lanatoside C. *n* = 3 biologically independent replicates per condition.

(B) Heatmap of proliferative marker expression in siScr or siTET2 macrophages treated with vehicle or Lanatoside C. *n* = 3 biologically independent replicates per condition.

Expression values in both heatmaps are shown as Z-scores.

Extended Data Fig. 3

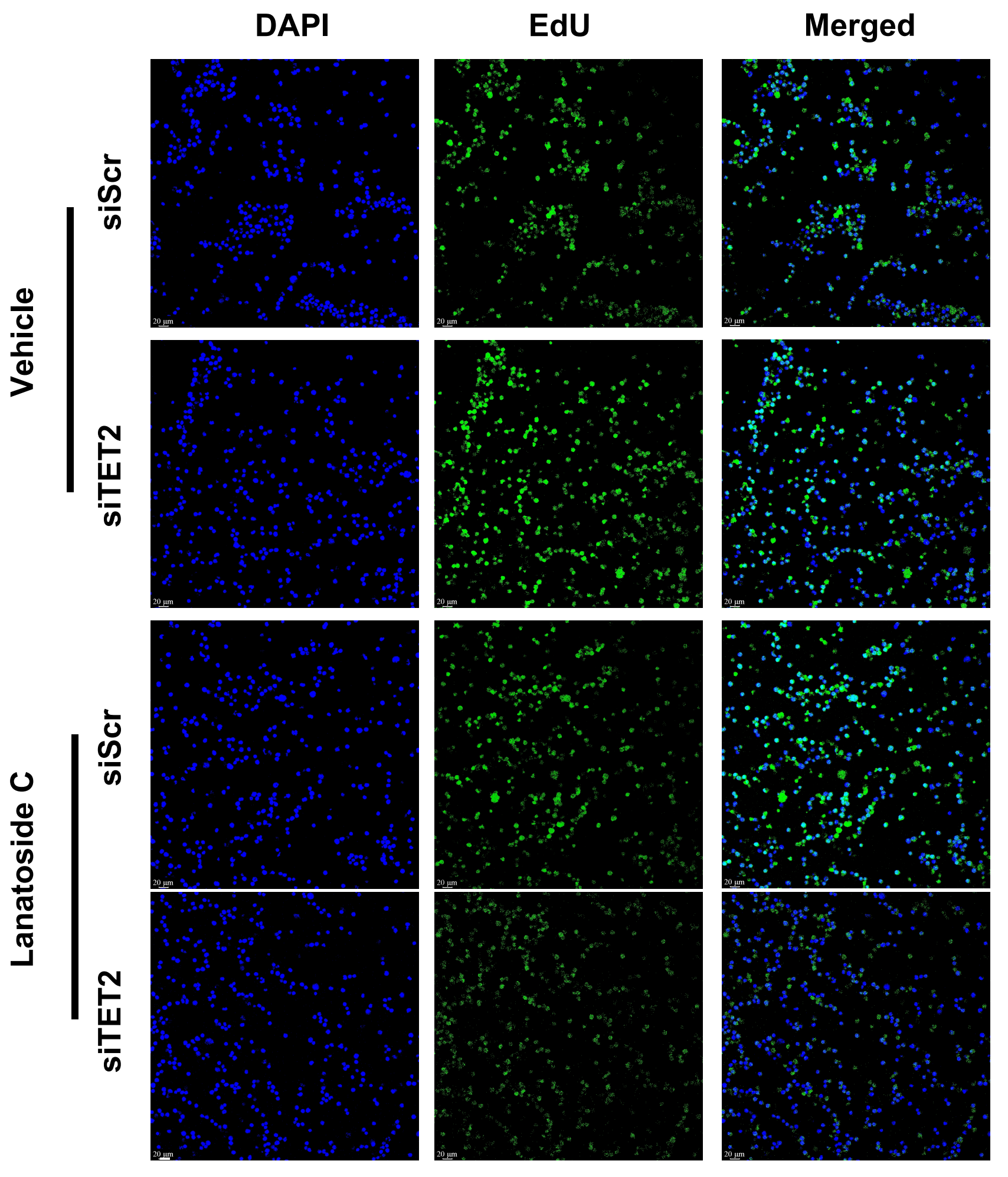

Extended Data Figure 3 | EdU staining in control and TET2-knockdown macrophages treated with vehicle or Lanatoside C.

Representative immunofluorescence images of siScr or siTET2 macrophages treated with vehicle (DMSO) or Lanatoside C and stained with DAPI (blue) and EdU (green).

Extended Data Fig. 4

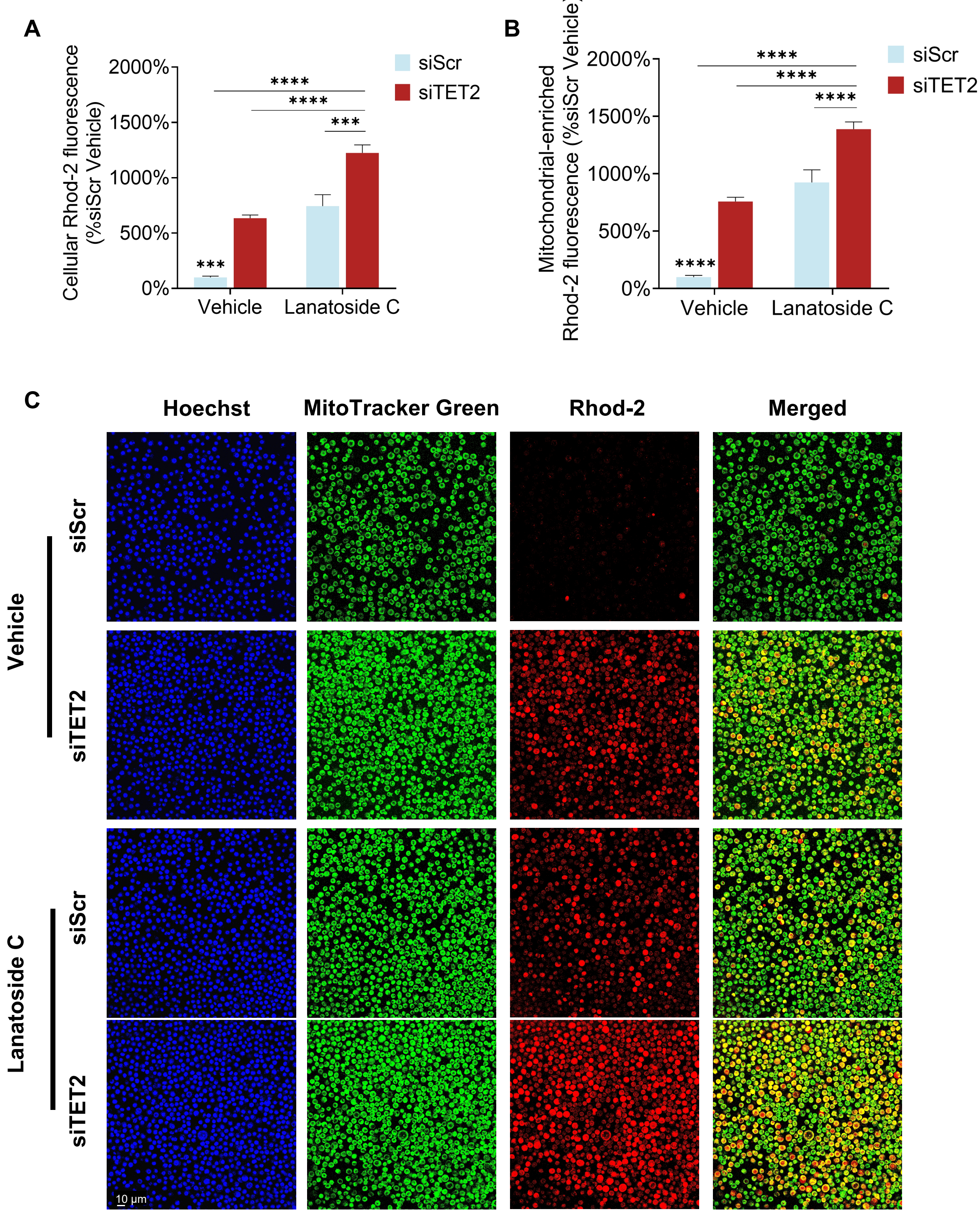

Extended Data Figure 4 | Lanatoside C enhances mitochondrial-enriched Rhod-2 Ca^{2+} signals in *TET2*-knockdown macrophages.

(A) Quantification of cellular Rhod-2 fluorescence in siScr and siTET2 THP-1-derived macrophages treated with vehicle or Lanatoside C. Rhod-2 fluorescence was quantified on a per-cell basis and normalized to the siScr vehicle group. Cell-level measurements were averaged within each biological replicate to generate a single replicate-level value before statistical analysis.

(B) Quantification of Rhod-2 fluorescence within MitoTracker Green-positive regions in macrophages treated as in (A), representing mitochondrial-enriched $Ca2^{+}$-sensitive Rhod-2 signal. Values were normalized to the siScr vehicle group. Measurements from individual cells were averaged within each biological replicate to generate a single replicate-level value before statistical analysis.

(C) Representative confocal fluorescence images of siScr and siTET2 macrophages treated with vehicle or Lanatoside C and labelled with MitoTracker Green (green), Rhod-2 (red), and Hoechst 33342 (blue). Merged images show the spatial overlap between Rhod-2 and MitoTracker Green signals. Scale bar, 10 µm.

Data are shown as mean ± SEM. $n = 6$ biologically independent replicates per condition. Statistical significance in (A,B) was assessed using two-way ANOVA followed by Tukey's multiple-comparisons test, with the indicated pairwise comparisons shown in the figure. $^{***}p < 0.001$, $^{****}p < 0.0001$.

Extended Data Fig. 5

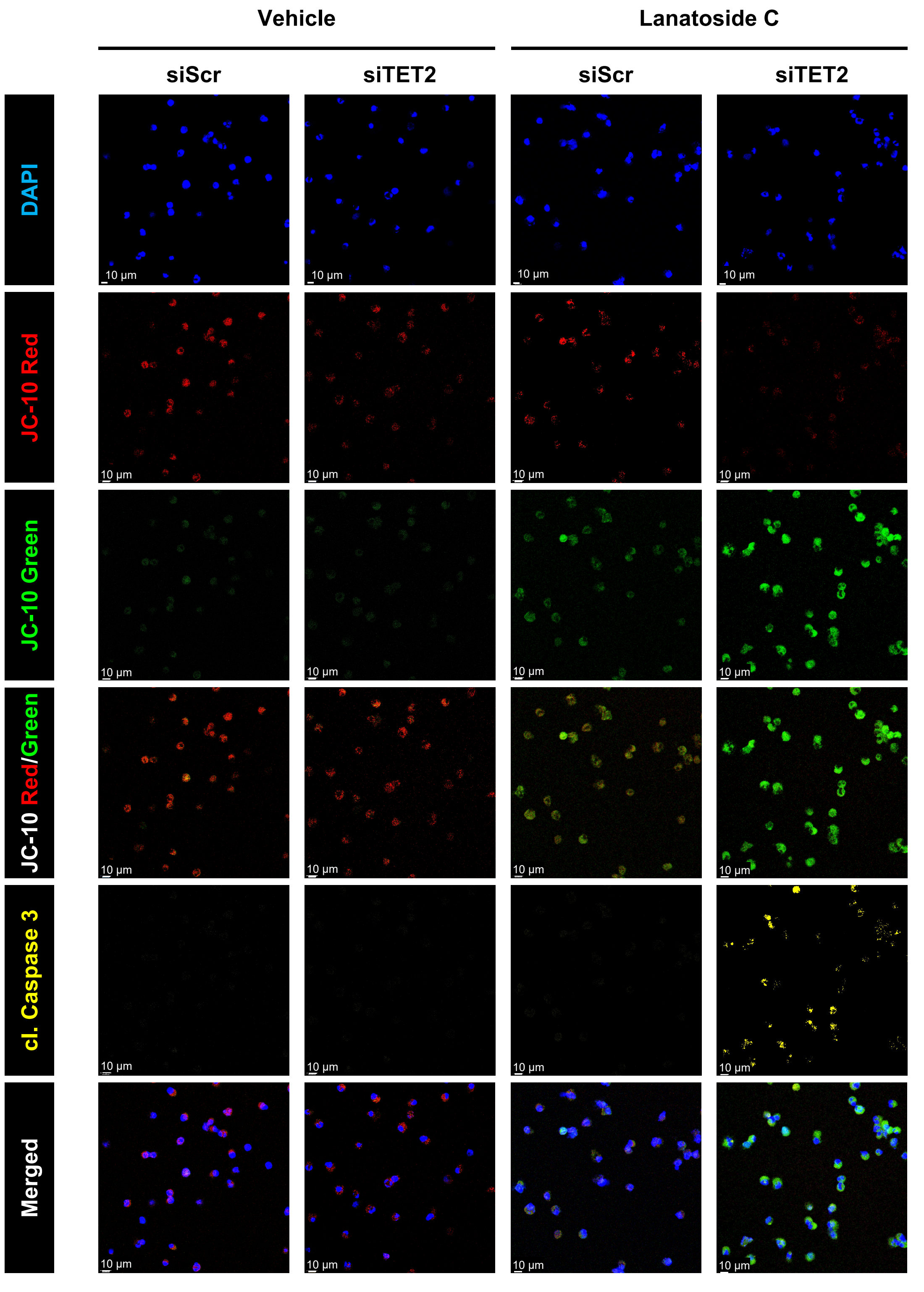

Extended Data Figure 5 | JC-10 and cleaved-Caspase-3 staining in control and TET2-knockdown macrophages.

Representative immunofluorescence images of siScr and siTET2 macrophages treated with vehicle (DMSO) or Lanatoside C and stained with DAPI (blue), JC-10 red (J-aggregates), JC-10 green (monomers), and cleaved Caspase-3 (yellow). Scale bar, 10 μm.

Extended Data Fig. 6

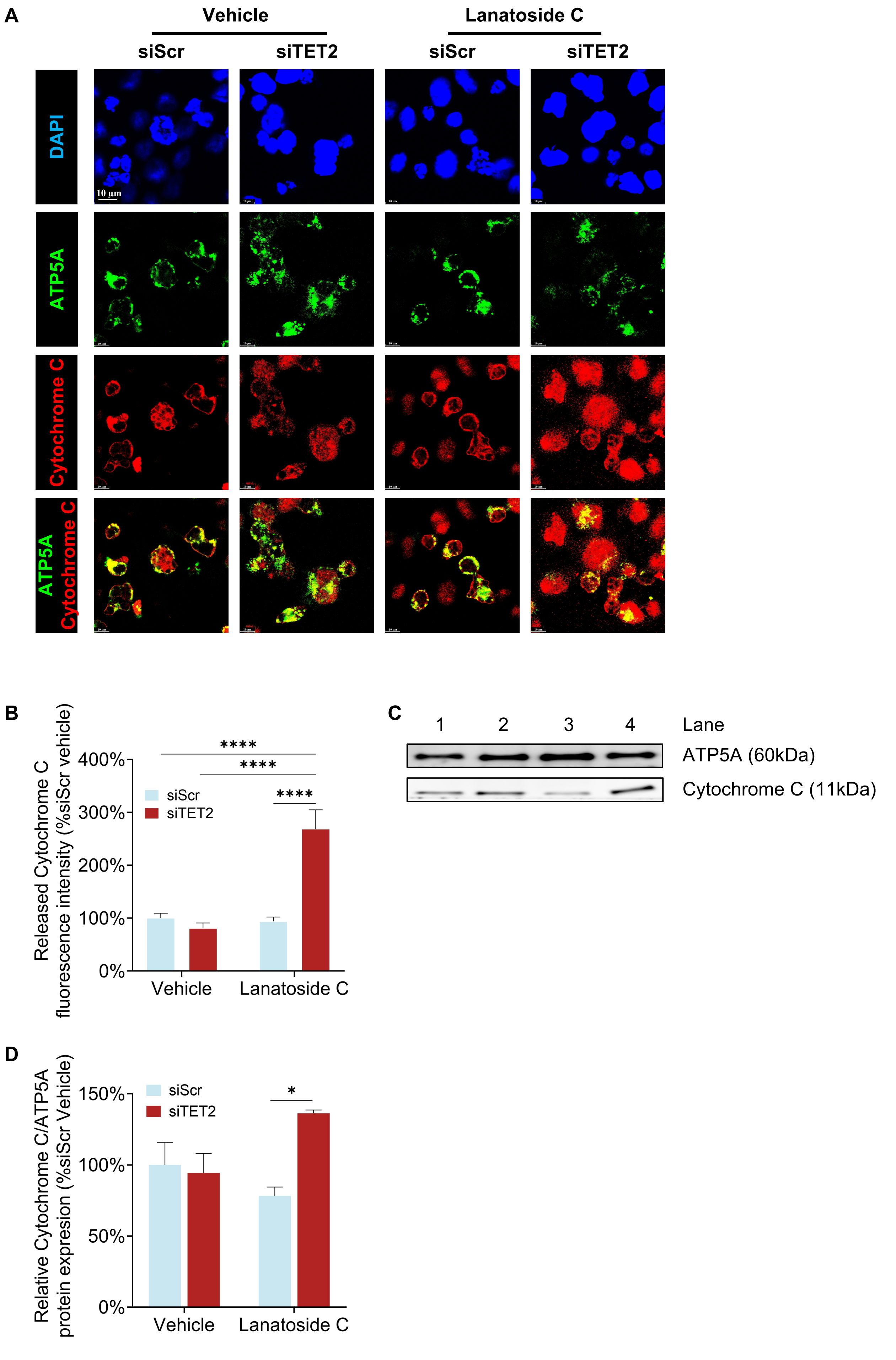

Extended Data Figure 6 | Cytochrome c redistribution in control and TET2-knockdown macrophages treated with Lanatoside C.

(A) Representative immunofluorescence images of siScr and siTET2 macrophages stained with DAPI (blue), ATP5A (green), and cytochrome c (red) after treatment with vehicle or Lanatoside C. Scale bar, 10 µm.

(B) Quantification of cytochrome c redistribution from mitochondria, assessed as cytochrome c fluorescence outside ATP5A-positive mitochondrial regions. $n = 8$ biologically independent replicates per condition.

(C) Representative Western blot of cytochrome c protein abundance in siScr and siTET2 macrophages treated with vehicle or Lanatoside C. ATP5A served as loading control. Lanes 1-4 represent siScr + vehicle, siTET2 + vehicle, siScr + Lanatoside C, and siTET2 + Lanatoside C, respectively.

(D) Densitometric quantification of cytochrome c protein abundance from the immunoblot analysis shown in (C). $n = 3$ biologically independent replicates per condition. ATP5A served as loading control.

Data are shown as mean ± SEM. Statistical significance in (B,D) was assessed using two-way ANOVA followed by Tukey's multiple-comparisons test, with the indicated pairwise comparisons shown in the figure. $^{*}p < 0.05$, $^{**}p < 0.01$, $^{***}p < 0.001$, $^{****}p < 0.0001$.

Extended Data Fig. 7

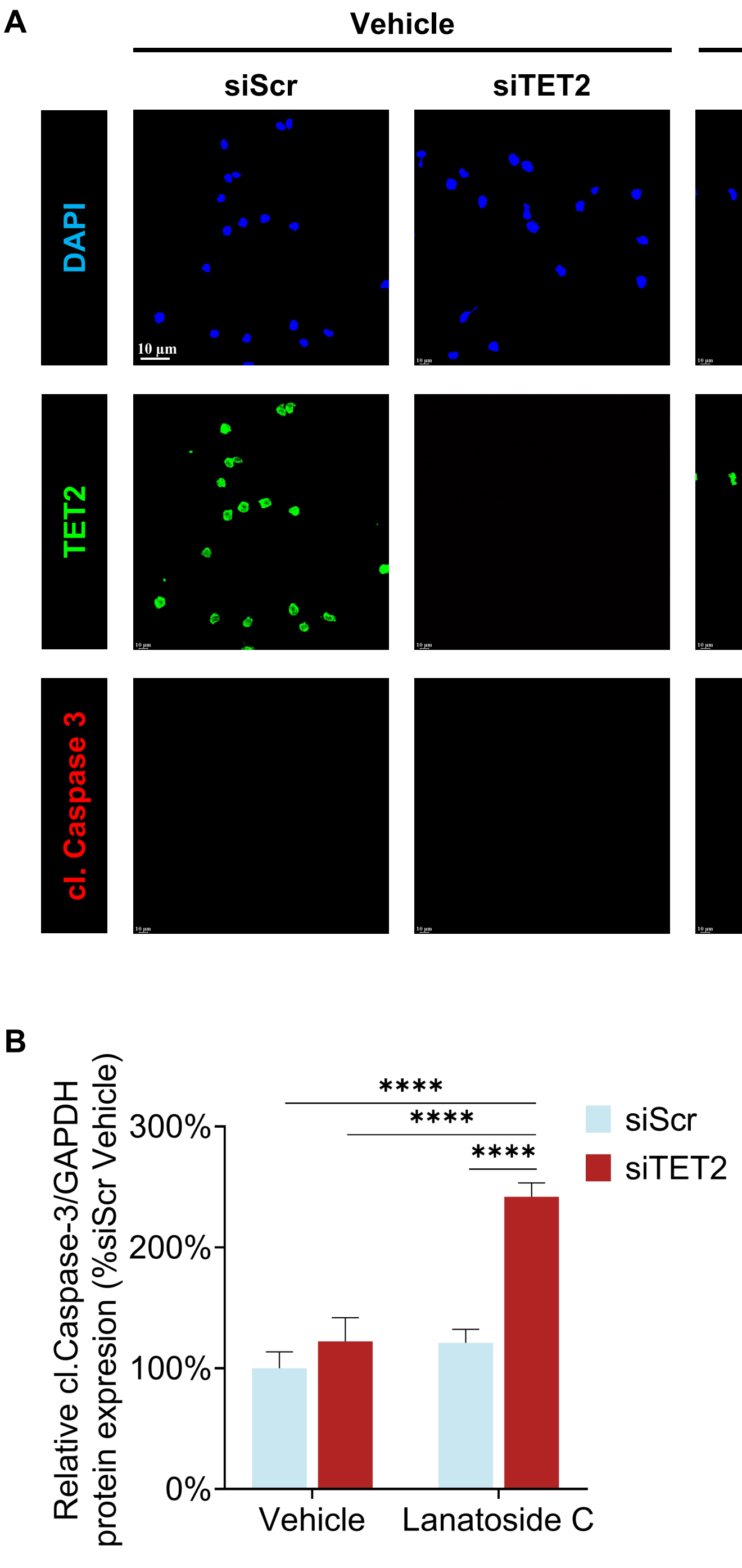

Extended Data Figure 7 | *TET2*-matched cleaved-Caspase-3 staining in control and TET2-knockdown macrophages treated with Lanatoside C.

(A) Representative immunofluorescence images of siScr and siTET2 macrophages treated with vehicle or Lanatoside C and stained for TET2 (green), cleaved-Caspase-3 (red), and DAPI (blue). Scale bar, 10 µm.

(B) Densitometric quantification of cleaved Caspase-3 protein abundance in siScr and siTET2 macrophages treated with vehicle or Lanatoside C. $n = 6$ biologically independent replicates per condition. GAPDH served as loading control.

Data are shown as mean ± SEM. Statistical significance in (B) was assessed using two-way ANOVA followed by Tukey's multiple-comparisons test, with the indicated pairwise comparisons shown in the figure. $^{*}p < 0.05$, $^{**}p < 0.01$, $^{***}p < 0.001$, $^{****}p < 0.0001$.

Extended Data Fig. 8

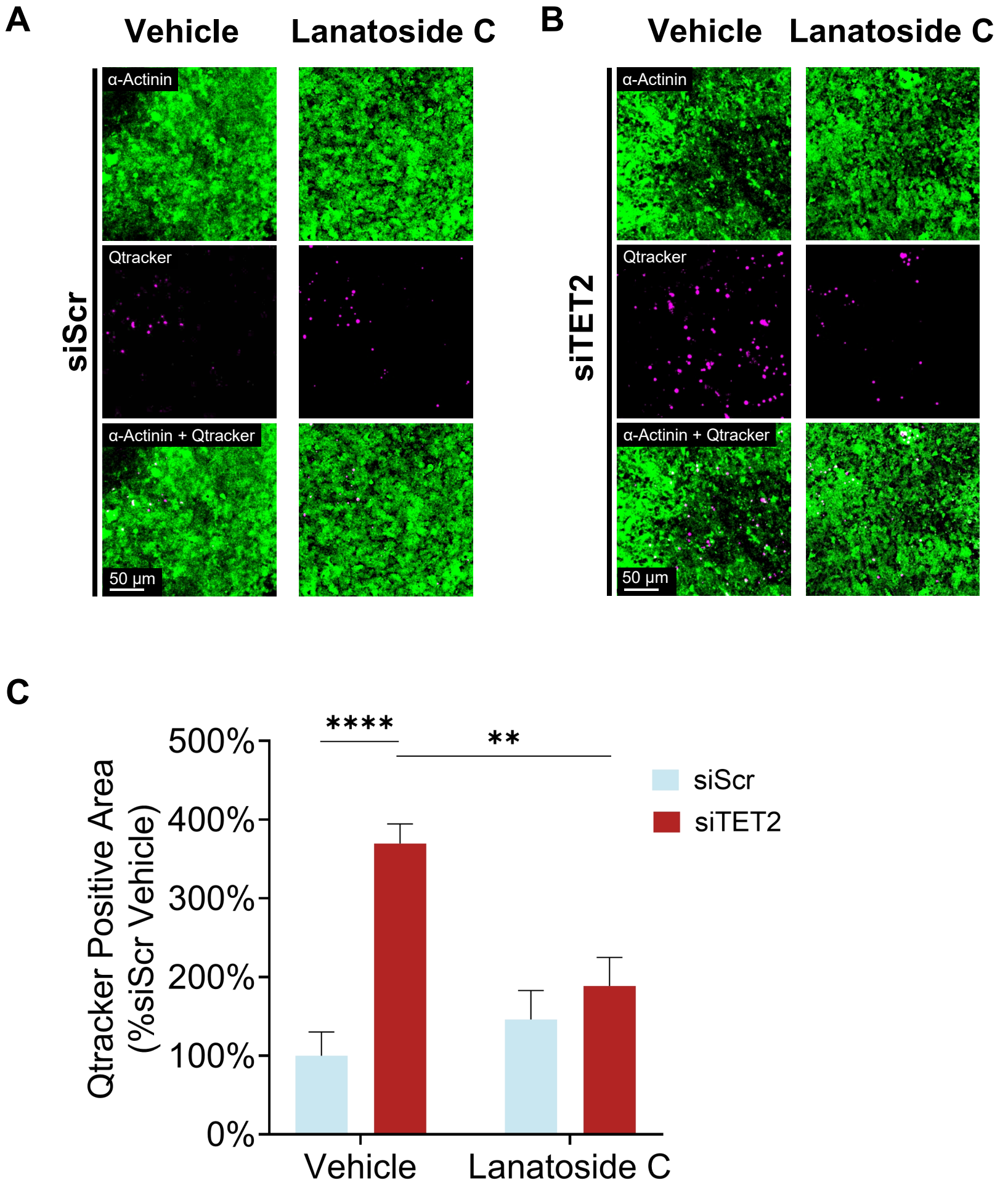

Extended Data Figure 8 | Macrophage infiltration in CDMs treated with vehicle or Lanatoside C.

(A, B) Representative immunofluorescence images of CDMs infiltrated by siScr (A) or siTET2 **(B)** macrophages and treated with vehicle or Lanatoside C. CDMs were stained with α-Actinin (green), and infiltrating macrophages with Qtracker (magenta). Scale bar, 50 µm.

(C) Quantification of macrophage-infiltrated areas in CDMs treated as in (A,B). $n = 6$ biologically independent samples per condition; quantification was performed using non-overlapping imaging areas.

Data are shown as mean ± SEM. For each biological replicate, measurements from non-overlapping imaging areas were averaged to generate a single replicate-level value before statistical analysis. Statistical significance in (C) was assessed using two-way ANOVA followed by Tukey's multiple-comparisons test, with the indicated pairwise comparisons shown in the figure. $^{*}p < 0.05$, $^{**}p < 0.01$, $^{***}p < 0.001$, $^{****}p < 0.0001$.

Extended Data Fig. 9

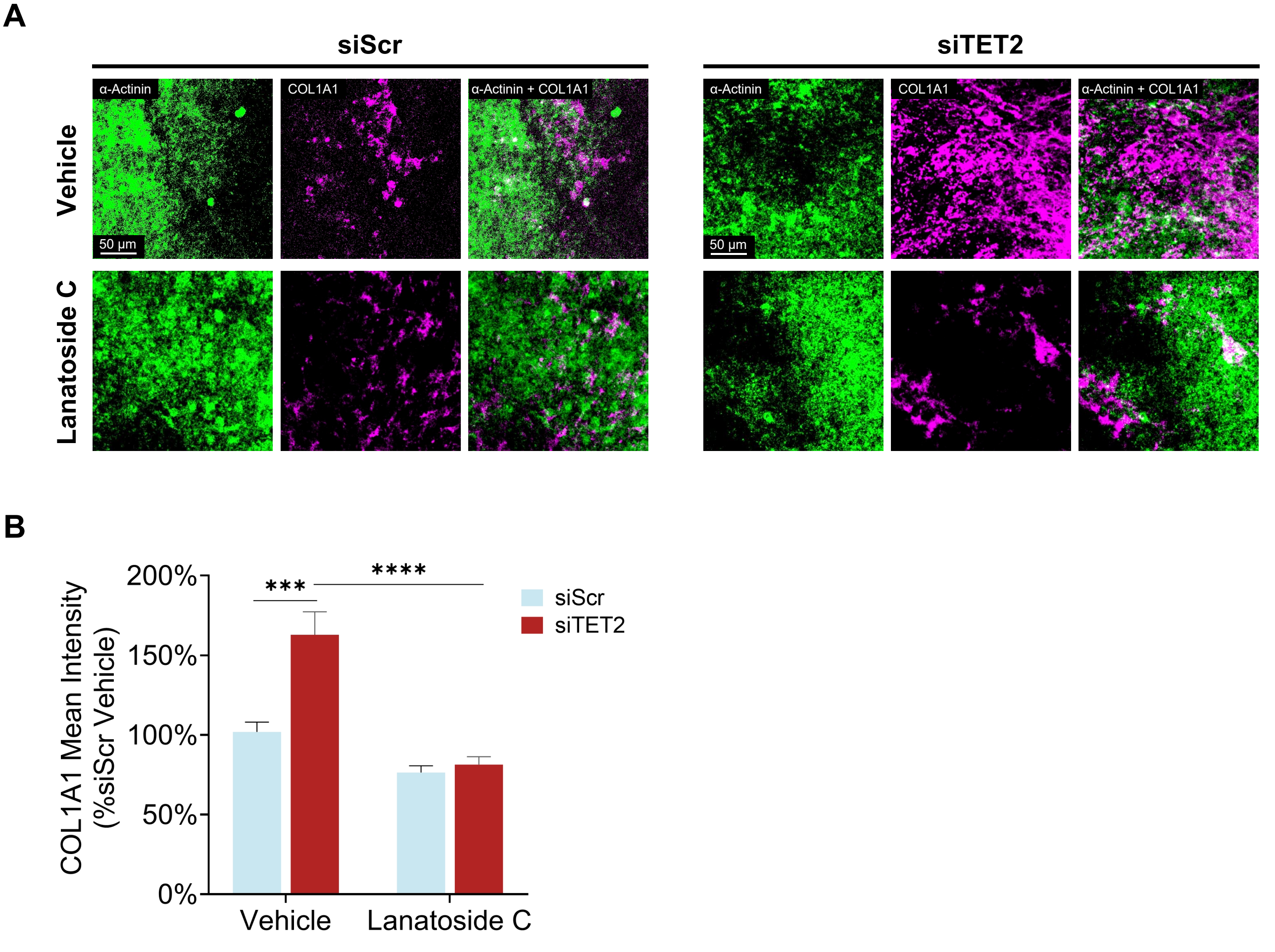

Extended Data Figure 9 | Lanatoside C reduces fibrosis in TET2-KD macrophage-infiltrated CDMs.

(A) Representative immunofluorescence images of CDMs infiltrated by siScr or siTET2 macrophages and treated with vehicle or Lanatoside C. CDMs were stained with α-Actinin (green) and COL1A1 (magenta). Scale bar, 50 µm.

(B) Quantification of mean COL1A1 fluorescence intensity in CDMs treated as in (A). $n = 6$ biologically independent samples per condition; quantification was performed using non-overlapping imaging areas.

Data are shown as mean ± SEM. For each biological replicate, measurements from non-overlapping imaging areas were averaged to generate a single replicate-level value before statistical analysis. Statistical significance in (B) was assessed using two-way ANOVA followed by Tukey's multiple-comparisons test, with the indicated pairwise comparisons shown in the figure. $^{*}p < 0.05$, $^{**}p < 0.01$, $^{***}p < 0.001$, $^{****}p < 0.0001$.

Extended Data Fig. 10

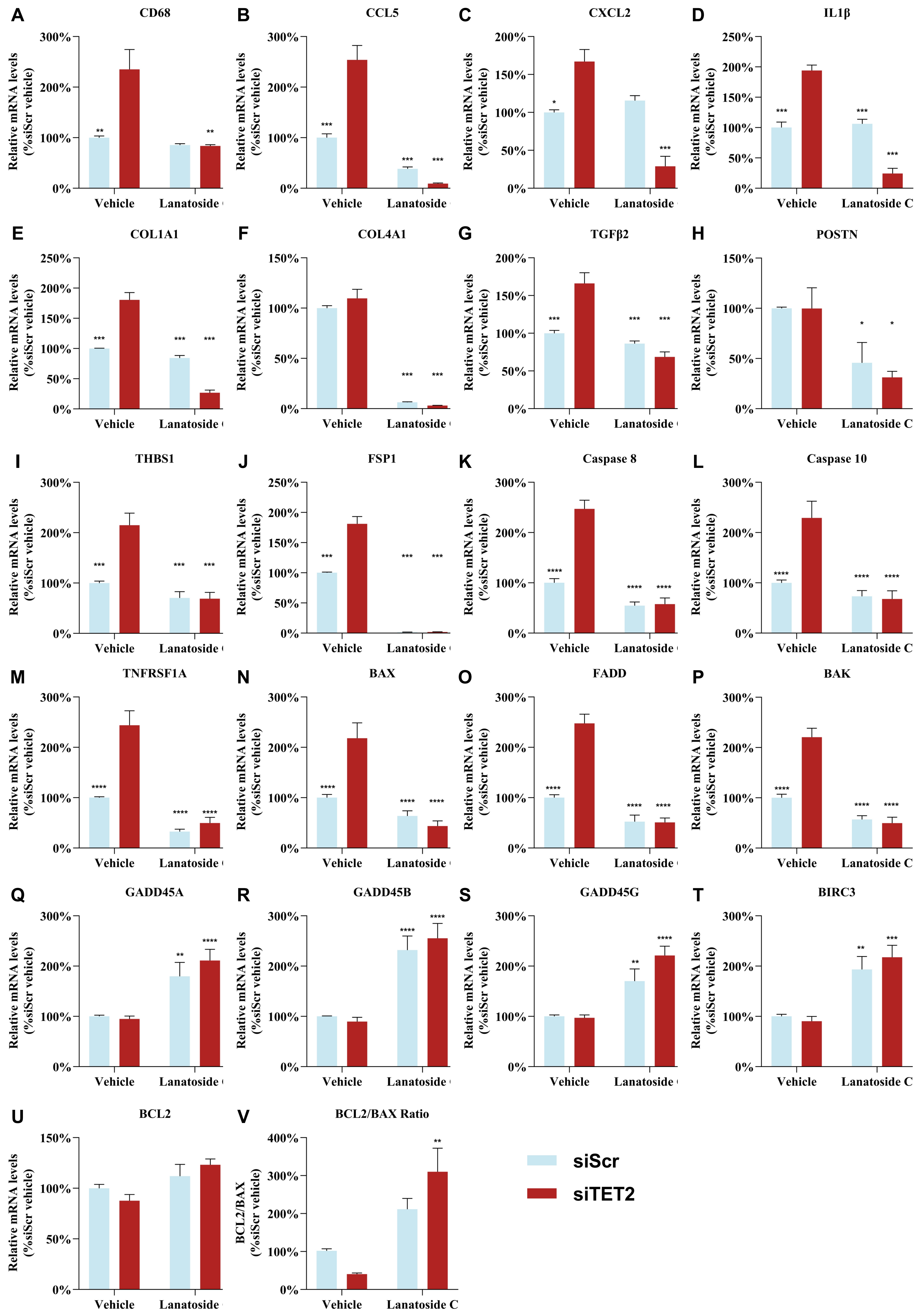

Extended Data Figure 10 | Expression of inflammation-, fibrosis-, and apoptosis-related genes in TET2-KD macrophage-infiltrated CDMs treated with vehicle or Lanatoside C.

(A to D) Expression of inflammatory genes.

(E to J) Expression of fibrosis genes.

(K to P) Expression of pro-apoptotic genes.

(Q to U) Expression of anti-apoptotic genes.

(V) BCL2/BAX expression ratio.

Data are shown as mean ± SEM (n = 4 biologically independent samples per condition). Statistical significance in (A-V) was assessed using two-way ANOVA followed by Tukey's multiple-comparisons test, with the indicated pairwise comparisons shown in the figure. $^{*}p < 0.05$, $^{**}p < 0.01$, $^{***}p < 0.001$, $^{****}p < 0.0001$.

Extended Data Fig. 11

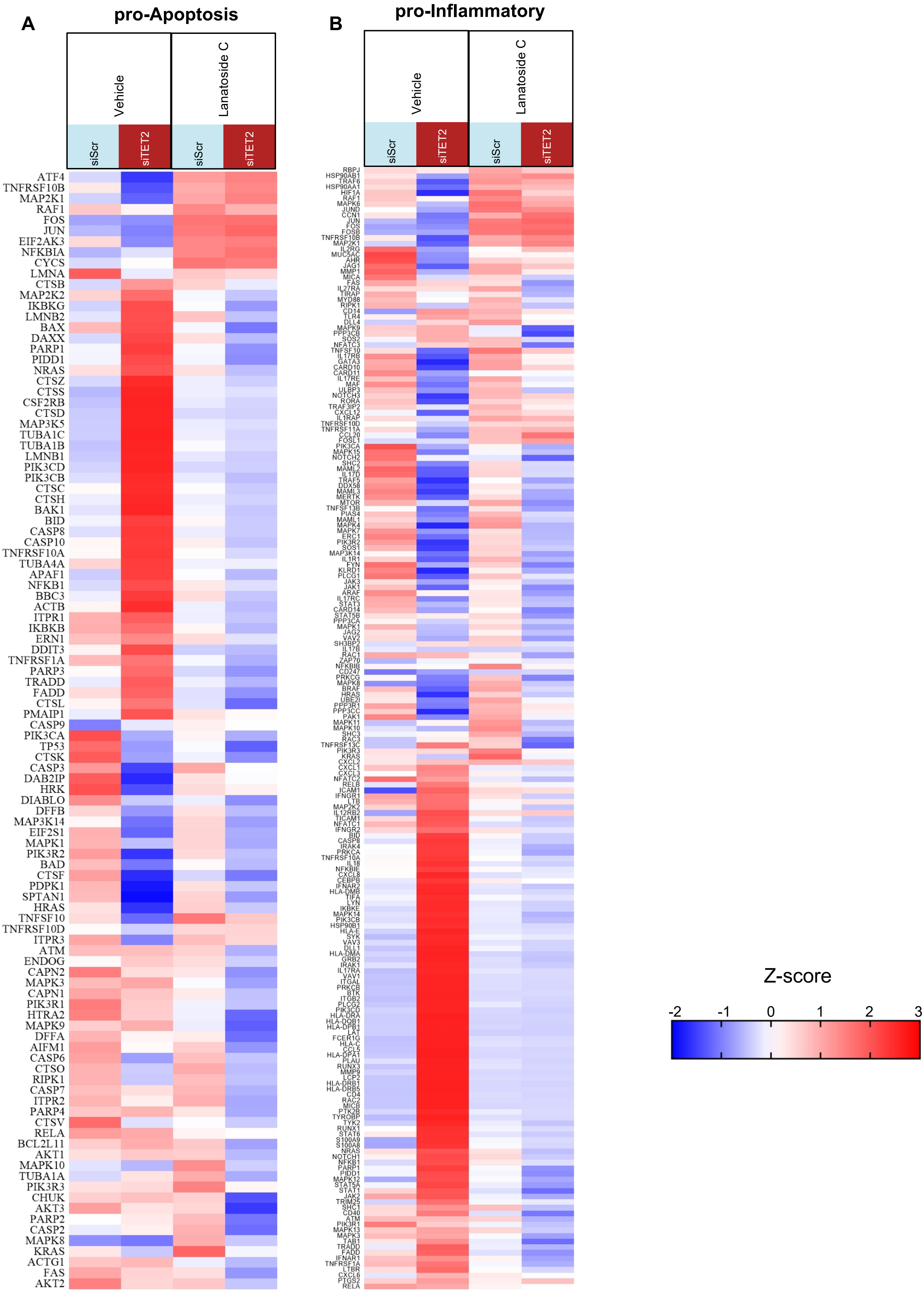

Extended Data Figure 11 | Extended RNA expression analysis of control and TET2-KD macrophage-infiltrated CDMs treated with Lanatoside C.

(A) Heatmap of pro-apoptotic markers derived from bulk RNA sequencing of CDMs infiltrated by siScr or siTET2 macrophages and treated with vehicle or Lanatoside C. *n* = 3 biologically independent replicates per condition, with 100 pooled CDMs per replicate.

(B) Heatmap of pro-inflammatory markers derived from bulk RNA sequencing of CDMs treated as in (A). *n* = 3 biologically independent replicates per condition, with 100 pooled CDMs per replicate.

Expression values in both heatmaps are shown as Z-scores.

Extended Data Fig. 12

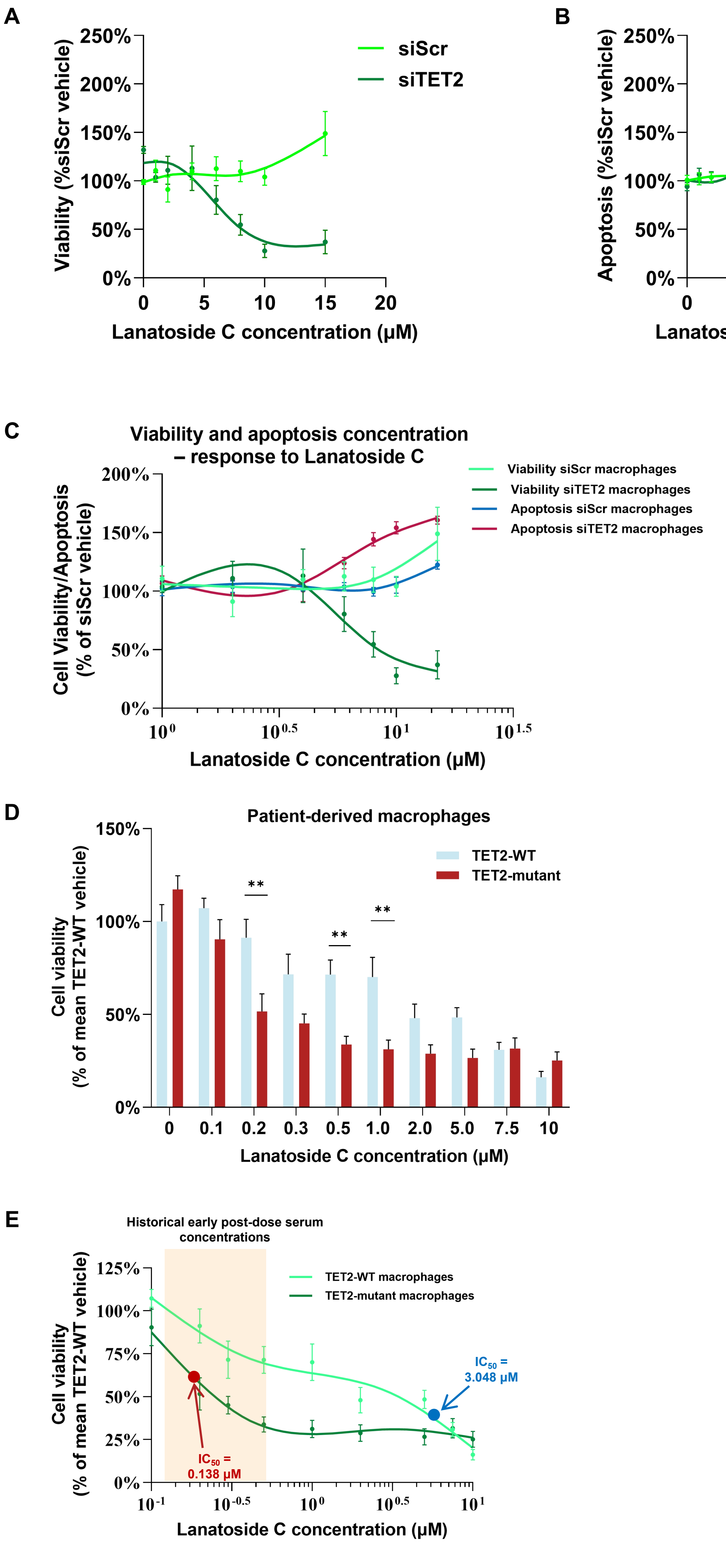

Extended Data Figure 12 | Differential Lanatoside C sensitivity in siTET2-transfected primary human macrophages and TET2-mutant patient-derived macrophages.

(A) Dose-response analysis of cell viability in primary human monocyte-derived macrophages transfected with siScr or siTET2 and treated with Lanatoside C (0, 1, 2, 4, 6, 8, 10 and 15 µM) for 48 h. Macrophages were derived from four independent human donors.

(B) Dose-response analysis of apoptosis in siScr- and siTET2-transfected primary human macrophages treated as in (A).

(C) Combined four-parameter logistic concentration-response fits of the viability and apoptosis data shown in (A,B), illustrating the differential Lanatoside C sensitivity associated with siTET2 transfection in primary human macrophages.

(D) Concentration-response analysis of cell viability in patient-derived macrophages stratified according to sequencing-confirmed TET2 mutation status and treated with Lanatoside C (0, 0.1, 0.2, 0.3, 0.5, 1.0, 2.0, 5.0, 7.5 and 10 µM) for 48 h. Values were expressed relative to the mean vehicle response of the *TET2*-WT comparator group, which was set to 100%. $n = 8$ independent individuals per group.

(E) Four-parameter logistic concentration-response fits of the patient-derived macrophage viability data shown in (D). The IC_{50} point estimate was 0.138 µM (95% CI, 0.106-0.177 µM) for *TET2*-mutant macrophages and 3.048 µM (95% CI, 0.517 µM to an upper limit that could not be estimated from the concentration range tested) for *TET2*-WT macrophages. The *TET2*-mutant estimate was therefore well bounded within the tested range, whereas the *TET2*-WT estimate was comparatively imprecise; the apparent greater-than-20-fold separation between the point estimates should consequently be interpreted cautiously. The shaded region indicates the approximate mean serum concentrations reported immediately after intravenous Lanatoside C administration (0.25 µg/mL; approximately 0.254 µM) and at 7.5 min after dosing (0.10 µg/mL; approximately 0.102 µM) in a historical human study.

Data are shown as mean ± SEM. The viability and apoptosis data in (A,B) were analysed separately using mixed-effects models with concentration, siRNA condition and their interaction as fixed effects and donor as a random effect. The patient-derived viability data in (D) were analysed using a mixed-effects model with concentration, TET2 mutation status and their interaction as fixed effects and participant as a random effect. Šídák-adjusted planned comparisons were used to compare the two conditions or groups at each concentration; the significance symbols shown in (A,B,D) represent these adjusted comparisons. Concentration-

response curves in (C,E) were fitted using four-parameter logistic regression as described in the Methods. For (A-C), $n = 4$ independent human donors; for (D,E), $n = 8$ independent individuals per group, with each donor or participant treated as one biological replicate. $^{*}p < 0.05$, $^{**}p < 0.01$, $^{***}p < 0.001$, $^{****}p < 0.0001$.

Extended Data Table 1. Compound library used for primary screen

Daurisoline	55-Dimethyloxazolidine-24-dione	Notoginsenoside R1	Trimethadione	Khellin	Ro 46-2005
LXR623	Chlorophyllin	Daphnetin	Hexamethonium Bromide	Praziquantel	Remodelin hydrobromide
LCZ696	Ciprofibrate	Ginsenoside Rg1	Digitoxin	Lacidipine	CVT313
Roniacol	Tripelennamine hydrochloride	Hyperoside	Buflomedil hydrochloride	Hydrochlorothiazide	Belnacasan
Propofol	Tanshinone I	Ginsenoside Rb1	Fendiline hydrochloride	Candesartan	Selexipag
Trans-Anethole	Isoferulic acid	Daidzin	Carvedilol phosphate	Captopril	GW 441756
Dixanthogen	Myricetrin	Ginsenoside Re	Nimodipine	Duloxetine hydrochloride	Daprodustat
Lupetidin	Adenosine disodium triphosphate	Forskolin	Minaprine dihydrochloride	Otilonium bromide	Midostaurin
Pargyline	Vinpocetine	Sodium Aescinate	Labetalol hydrochloride	Indapamide	Lomerizine hydrochloride
Vitamin E	Ruxolitinib (INCB-18424) phosphate	Genipin	Riluzole	Olmesartan Medoxomil	Valsartan
Nikethamide	2-Bromo-6-methoxynaphthalene	Atorvastatin Calcium	Ticlopidine hydrochloride	Aliskiren hemifumarate	Guanabenz Acetate
Clofibrate	Nicotinic acid hydrazide	Potassium canrenoate	Cinepazide maleate	Rapamycin	Tabersonine
Pempidine	Hesperetin	Atopaxar Hydrobromide	Sildenafil citrate	Chlorpheniramine maleate	Dihydralazine sulphate
Carvedilol	Luteolin	Vorapaxar sulfate	Xylocaine	Diclofenac sodium	Delapril Hydrochloride
Ropivacaine hydrochloride	Triflusal	Clinofibrate	Octopamine hydrochloride	Deferidone	Disodium clodronate tetrahydrate
Probenecid	Lisinopril dihydrate	Benidipine hydrochloride	Nefopam hydrochloride	Rosuvastatin	Manidipine
Proflavine Hemisulfate	Naftopidil	Astaxanthin	Sertraline hydrochloride	Chitosan	Etilefrine hydrochloride
Cilnidipine	Ursodeoxycholic acid	U104	Metoprolol tartrate	Y-27632 dihydrochloride	Verbascoside
Menadione	Gabapentin	Ambroxol hydrochloride	Abbolactone	Articaine HCl	U73122
Glucosamine	Pranlukast	Eburnalritardo	Doxofylline	Omeprazole sulfide	PD123319
Dabigatran etexilate	Lamotrigine	Synephrine	Zinc pyrithione	Guanfacine hydrochloride	Sotrastaurin
Oxybuprocaine hydrochloride	Mevastatin	IBMX	Cyclandelate	Felypressin	Isosteviol
Oxcarbazepine	Stugeron	Diazoxide	L-(-)-α-Methyldopa	Amoxapine	Go 6983
Atropine sulfate monohydrate	Pravastatin sodium	Tanshinone IIA sulfonate sodium	Tolperisone hydrochloride	Telmisartan	Gamma-Oryzanol

Pramoxine hydrochloride	Lansoprazole	Ginkgolide B	Phenylindione	Midodrine hydrochloride	Chelerythrine chloride
Phenylephrine hydrochloride	Chlorothiazide	Ibudilast	Tioxolone	Pargyline hydrochloride	Mozavaptan
Edetate Trisodium	Omeprazole	Citalopram HBr	Theophylline monohydrate	Desvenlafaxine	STO-609 acetate
Ellagic acid	Carbaglu	Amrinone	Manidipine dihydrochloride	Metolazone	PS1145
Cilostazol	DL-Carnitine	Moxonidine	Lidocaine Hydrochloride hydrate	Hydralazine hydrochloride	Propanoic acid
Eprosartan Mesylate	Ethosuximide	Nicorandil	Paeoniflorin	Enalapril Maleate	HTHQ
Phenytoin sodium	Pindolol	Dantrolene sodium salt	Bengenin	Irbesartan	YM58483
Methylene Blue trihydrate	Urea	Domperidone	Metyrapone	Dypyridamole	CP 640186
Mecarbinate	Diosmin	Trapidil	Amiloride hydrochloride dihydrate	Trazodone hydrochloride	VTP27999
Cinchonine	6-Aminocaproic acid	Eplerenone	Ranolazine dihydrochloride	Deferoxamine Mesylate	Secoisolariciresinol diglucoside
Ethamsylate	Topiramate	Roflumilast	Ropivacaine	Glyburide	Quercetin Dihydrate
Sodium Nitroprusside	Adenosine 5'-monophosphate monohydrate	Nifekalant hydrochloride	Fluoxetine hydrochloride	Beta-Carotene	Levobupivacaine HCl
Ferrous fumarate	Gastrodin	Treprostinil Sodium	Vardenafil	Lenalidomide	Ruboxistaurin hydrochloride
Heptaminol hydrochloride	Cordycepin	Ruxolitinib	Indazole	Dopamine hydrochloride	GW501516
Oxethazaine	Rotundine	Momelotinib	Ferulic Acid	Ramipril	Darapladib
Pentoxifylline	Proanthocyanidins	Necrostatin-1	Daidzein	Rebeprazole sodium	R406
Enoxolone	Scoparone	Ilaprazole sodium	Dorzolamide hydrochloride	Tolazoline hydrochloride	NU2058
Miltefosine	Asiatic acid	6-BIO	Novobiocin Sodium	Aminophylline	Turofexorate Isopropyl
Primidone	Limonin	Dinaciclib	Dequalinium chloride	Methacholine Chloride	Calpeptin
Bupivacaine hydrochloride	Puerarin	Everolimus	Methoxamine hydrochloride	Mirabegron	3',4'-Dihydroxyacetophenone
Procainamide hydrochloride	Pterostilbene	Levobupivacaine	Trimetazidine dihydrochloride	Tipiracil hydrochloride	Rilmenidine Phosphate
Clofibric acid	Butyl 4-aminobenzoate	Methazolamide	Clevidipine butyrate	Edoxaban Tosylate Monohydrate	Dofetilide
Nicotinic acid N-oxide	Benfluorex hydrochloride	Fosinopril sodium	Perindopril erbumine	Ponatinib	Vesnarinone
Urapidil hydrochloride	Benzocaine	Isosorbide dinitrate	Tranylcypromine (2-PCPA) hydrochloride	RGB-286638 free base	SRT2104
Alfuzosin hydrochloride	Reserpine	Fluvastatin sodium salt	Mafenide hydrochloride	Edoxaban	Temocapril HCl

Molsidomine	(-)-Sparteine sulfate pentahydrate	Rosuvastatin calcium	R-(+)-Lansoprazole	Pomalidomide	AZD6482
Diltiazem hydrochloride	Benzthiazide	Ambrisentan	Hesperidin	BX795	Ibutilide Fumarate
Nitrendipine	Tolfenamic Acid	Regadenoson	Trimipramine maleate	Eniporide	Losartan Carboxylic Acid
Azelnidipine	Quinapril hydrochloride	Tiplaxtinin	Mexiletine hydrochloride	Pirinixic Acid	NQ301
Trichlormethiazide	Flunarizine dihydrochloride	WHI-P180	Azilsartan	KenPaullone	Lercanidipine hydrochloride
1-Ethyl-2-benzimidazolinone	Propitocaine hydrochloride	Omecamtiv mecarbil	Ketanserin	Anacetrapib	ACY738
Brinzolamide	Acetazolamide	Apabetalone	Nafronyl oxalate	Indacaterol	CPI637
(±)-Bisoprolol hemifumarate	Dicumarol	Macitentan	Theophylline	SC514	Dalcetrapib
Felodipine	Etofylline	PF562271	Chlortalidone	Guanethidine Monosulfate	Acecainide hydrochloride
Anagrelide	Furosemide	KI8751	Milrinone	Etofibrate	WAY 200070
Pantoprazole Sodium Hydrate	Bezafibrate	Galunisertib	Lidocaine hydrochloride	Ivabradine hydrochloride	Rhapontigenin
Argatroban	Ademine	AZD5438	Nifedipine	Rufinamide	(+)-Usniacin
Nisoldipine	L(-)-Carnitine	Roxadustat	Fenofibrate	Lercanidipine	Bosentan
Urapidil	Carbazochrome sodium sulfonate	SL327	Orphenadrine hydrochloride	Tirofiban hydrochloride monohydrate	Enalaprilat Dihydrate
Nylidrin hydrochloride	Adenosine	Trans-Tranilast	Bendazol	Levosimendan	AZD1283
Amiloride hydrochloride	Propafenone	Palosuran	Nortriptyline hydrochloride	Pimobendan	MGL3196
Ticagrelor	Atomoxetine hydrochloride	FG2216	Disopyramide	VX702	12346-O-Pentagalloylglucose
Benazepril hydrochloride	Noradrenaline bitartrate	Avasimibe	Permethrin	Torcetrapib	Vorapaxar
Levetiracetam	Quercetin	Apixaban	Hydroquinidine	Cariporide	Dextrorphan tartrate
Terazosin hydrochloride	Carbamazepine	Ramatroban	N-Acetyl-5-hydroxytryptamine	Losmapimod	(S)-Amlodipine Besylate (103129-82-4(free base))
Loperamide hydrochloride	Mepivacaine hydrochloride	Camostat mesilate	(±) Clopidogrel hydrogen sulfate	LY2874455	PF06282999
Seratrodast	Phenytoin	Gandotinib	(S)-(+)-Clopidogrel hydrogen sulfate	Ro 3306	MD 39-AM
Thalidomide	Isradipine	LY2811376	Aceglutamide	TAS301	XL652
Methyl hesperidin	Amlodipine	Rostafuroxin	Racecadotril	Taxifolin	Coelenterazine
Prasugrel	Phenazopyridine hydrochloride	WHI-P131	Rivaroxaban	Nafamostat mesylate	VAS2870

Proparacaine hydrochloride	Dyclonine hydrochloride	Varespladib	Bendroflumethiazide	Cinaciguat	Ranolazine
Amlodipine Besylate	Tadalafil	Purvalanol A	Trimebutine maleate	Fasudil hydrochloride	Escitalopram Oxalate
Acecainide	Fenofibric acid	BTB06584	Tetracaine hydrochloride	Fimasartan	Lificiguat
Mepiroxol	Capecitabine	AT 7519 hydrochloride salt	Ibandronate sodium	Fedratinib	Sarpogrelate hydrochloride
Chromocarbe	Methocarbamol	Ro 48-8071 fumarate	Nicardipine hydrochloride	Efonidipine	Desidustat
Quinidine hydrochloride	Torasemide	Ro 61-8048	Lovastatin	Tolvaptan	Lanatoside C
Zonisamide	Pentetrazol	CH5183284			
Escin	Nilvadipine	BIBS 39			
Metaraminol bitartrate	Trifluridine	Ro 31-8220 Mesylate			

Extended Data Table 2. siRNA sequences

Name	Sequences
ON-TARGETplus Non-targeting Control Pool	UGGUUUACAUGUCGACUAA
ON-TARGETplus Non-targeting Control Pool	UGGUUUACAUGUUUUCUGA
ON-TARGETplus Non-targeting Control Pool	UGGUUUACAUGUUGUGUGA
ON-TARGETplus Non-targeting Control Pool	UGGUUUACAUGUUUUCCUA
ON-TARGETplus Human TET2 siRNA	ACAAGAAAGUAGAGGGUAU
ON-TARGETplus Human TET2 siRNA	ACACCUAGUUUCAGAGAAU
ON-TARGETplus Human TET2 siRNA	CCUCAGAAUAAUUGUGUGA
ON-TARGETplus Human TET2 siRNA	CAGCAAAGGUACUUGAUAC

Extended Data Table 3. Primer sequences for qPCR

Name	Forward	Reverse
TET2	GTGAGGCTGCAGTGATTGTG	GATTGGTGAGCGTGCCGTAT
IL1β	AGCTACGAATCTCCGACCAC	CGTTATCCCATGTGTCGAAGAA
IL18	TCTTCATTGACCAAGGAAATCGG	TCCGGGGTGCATTATCTCTAC
CCL5	GGAAAGATTTTGTTGTTGTT	AGTCACCTACAATTCAAGAC
CXCL2	GGGCAGAAAGCTTGTCTCAA	GCTTCCTCCTTCCTTCTGGT
CD68	CTTCTCTCATTCCCCTATGGACA	GAAGGACACATTGTACTCCACC
MerTK	GTGCAGCGTTCAGACAATGG	TCGATGTAGATGGGATCAGACAC
NPPA	CAACGCAGACCTGATGGATTT	AGCCCCCGCTTCTTCATTC
NPPB	TGGAAACGTCCGGGTTACAG	CTGATCCGGTCCATCTTCCT
FSP1	CAGAACTAAAGGAGCTGCTGACC	CTTGGAAGTCCACCTCGTTGTC
HPRT	CCTGGCGTCGTGATTAGTGAT	AGACGTTCAGTCCTGTCCATAA
CASP8	GACAGAGCTTCTTCGAGACAC	CCTGAGTGAGTCTGATCCACAC
CASP10	AGAAACCTGCTCTACGAACTGT	GGGAAGCGAGTCTTTCAGAAG
TNFRSF1A	TCACCGCTTCAGAAAACCACC	GGTCCACTGTGCAAGAAGAGA
BAX	CCCGAGAGGTCTTTTTCCGAG	CCAGCCCATGATGGTTCTGAT
FADD	GTGGCTGACCTGGTACAAGAG	GGTAGATGCGTCTGAGTTCCAT
BAK	ATGGTCACCTTACCTCTGCAA	TCATAGCGTCGGTTGATGTCG
GADD45A	GAGAGCAGAAGACCGAAAGGA	CAGTGATCGTGCGCTGACT
GADD45B	TGCTGTGACAACGACATCAAC	GTGAGGGTTCGTGACCAGG
GADD45G	CAGATCCATTTTACGCTGATCCA	TCCTCGCAAAACAGGCTGAG
BCL2	AGGACTATTCGGCAGAGTACAT	TGATTCCAGGGTATTCCTCCTC
BIRC3	TTTCCGTGGCTCTTATTCAAACT	GCACAGTGGTAGGAACTTCTCAT

Extended Data Table 4. Antibody list

Name	Company	Ref.
anti-α-Actinin	Sigma-Aldrich	A7811-.2ML
Anti-cleaved Caspase-3	Abcam	Ab32042
Anti-PCM1	Sigma-Aldrich	HPA023374
Anti-CD68	Invitrogen	MA1-80133
Anti-CD68	Abcam	ab233172
Pan neuronal marker	Merck	MAB2300
VE-Cadherin	Cell Signaling	#2500
DAPI	Abcam	ab228549
Anti-alpha smooth muscle Actin	Abcam	ab202295
Alexa Flour 488	Invitrogen	A11017
Alexa Flour 555	Invitrogen	A21425
Phalloidin 647	Abcam	ab176759
Phalloidin 488	Abcam	ab176753
Anti-COL1A1 antibody	Sigma-Aldrich	HPA011795
Anti-ATP5A antibody	Abcam	ab14748
Anti-Cytochrome c antibody	Abcam	ab90529
Anti-GAPDH antibody	Cell Signaling	5174S
Goat Anti-Mouse IgG	Abcam	ab205719
Goat Anti-Rabbit IgG	Abcam	ab6721
Anti-TET2 Antibody - C-Terminus	Cell Signaling	#45010
Anti-TET2 Antibody - N-terminal	Abcam	ab230358
Anti-CD68 antibody [FA-11]	Abcam	ab53444

Anti-TET2 antibody	Invitrogen	MA5-31442
